# The Oxidation-Induced Autofluorescence Hypothesis: Red Edge Excitation and Implications for Metabolic Imaging

**DOI:** 10.3390/molecules25081863

**Published:** 2020-04-17

**Authors:** Alexey N. Semenov, Boris P. Yakimov, Anna A. Rubekina, Dmitry A. Gorin, Vladimir P. Drachev, Mikhail P. Zarubin, Alexander N. Velikanov, Juergen Lademann, Victor V. Fadeev, Alexander V. Priezzhev, Maxim E. Darvin, Evgeny A. Shirshin

**Affiliations:** 1 Faculty of Physics, M.V. Lomonosov Moscow State University, 1-2 Leninskie Gory, Moscow 119991, Russia; semenov@physics.msu.ru (A.N.S.); bp.jakimov@physics.msu.ru (B.P.Y.); rubekina.aa14@physics.msu.ru (A.A.R.); Victor_fadeev@mail.ru (V.V.F.); avp2@mail.ru (A.V.P.); 2 Center for Photonics and Quantum Materials, Skolkovo Institute of Science and Technology, Skolkovo Innovation Center, Nobel st., Building 3, Moscow 121205, Russia; d.gorin@skoltech.ru (D.A.G.); V.Drachev@skoltech.ru (V.P.D.); 3 Department of Physics, University of North Texas, Denton, TX 76203, USA; 4 International Intergovernmental Organization Joint Institute for Nuclear Research 6 Joliot-Curie St., Dubna, Moscow 141980, Russia; mikzart@gmail.com; 5 Faculty of Biology, M.V. Lomonosov Moscow State University, 1-12 Leninskie Gory, Moscow 119234, Russia; av-bioem@mail.ru; 6 Department of Dermatology, Venerology and Allergology, Center of Experimental and Applied Cutaneous Physiology, Charité–Universitäts medizin Berlin, Corporate Member of Freie Universität Berlin, Humboldt-Universität zu Berlin, and Berlin Institute of Health, Charitéplatz 1, 10117 Berlin, Germany; juergen.lademann@charite.de (J.L.); maxim.darvin@charite.de (M.E.D.); 7 Institute of Spectroscopy of the Russian Academy of Sciences, Fizicheskaya Str., 5, Troitsk, Moscow 108840, Russia

**Keywords:** autofluorescence imaging, red-edge excitation, NIR autofluorescence, endogenous fluorophores, oxidation products, ultraviolet irradiation, keratinocytes

## Abstract

Endogenous autofluorescence of biological tissues is an important source of information for biomedical diagnostics. Despite the molecular complexity of biological tissues, the list of commonly known fluorophores is strictly limited. Still, the question of molecular sources of the red and near-infrared excited autofluorescence remains open. In this work we demonstrated that the oxidation products of organic components (lipids, proteins, amino acids, etc.) can serve as the molecular source of such red and near-infrared excited autofluorescence. Using model solutions and cell systems (human keratinocytes) under oxidative stress induced by UV irradiation we demonstrated that oxidation products can contribute significantly to the autofluorescence signal of biological systems in the entire visible range of the spectrum, even at the emission and excitation wavelengths higher than 650 nm. The obtained results suggest the principal possibility to explain the red fluorescence excitation in a large class of biosystems—aggregates of proteins and peptides, cells and tissues—by the impact of oxidation products, since oxidation products are inevitably presented in the tissue. The observed fluorescence signal with broad excitation originated from oxidation products may also lead to the alteration of metabolic imaging results and has to be taken into account.

## 1. Introduction

Autofluorescence (AF), i.e., intrinsic fluorescence of endogenous fluorophores, is extensively used in a range of biomedical applications [1]. Being non-invasive and label-free method, it has proven to be an indispensable tool for intraoperative diagnostics (fluorescence-guided surgery) [2], analysis of biofluids (blood [3,4], saliva [5], urine [6,7]), optical biopsy [8,9,10] etc. AF analysis is usually performed on a phenomenological basis, which means discrimination between objects (e.g., normal and pathological tissues, or between different components of the tissue) basing on differences in their AF intensity, spectral band shape or lifetime [11,12] and photobleaching rate [13]. Understanding of the photophysical mechanisms of fluorescence signal formation remains an important, yet challenging task. Despite the complexity of molecular composition of bio tissues, the list of endogenous fluorophores to refer is rather limited and thoroughly reviewed [11,14,15]. Namely, it is a common knowledge that tissue AF in the visible spectral range mainly originates from NAD(P)H, flavins, structural proteins (collagen, keratin, elastin), porphyrins and pigments (lipofuscin and melanin). The classical example of AF application is the use of NAD(P)H and FAD fluorescence parameters (intensity and lifetime) to reveal the metabolic state of living cells. This approach, known as optical metabolic imaging, allows for non-invasive studies of cells differentiation and response to external agents such as chemotherapy [16]. However, fluorescence formation in cells and tissues is more complex than it could be suggested by the list of standard fluorophores, and in some cases the origin of fluorescence is debatable. That is, AF in the red and near-infrared (NIR) regions of the spectrum has been reported for a wide range of systems [15,16,17,18,19,20]. On the one hand, this signal is a negative factor complicating analysis in certain types of experiments, e.g., when it is overlapped with a signal from exogenous probe or when it masks the Raman scattering signal. In the latter case, excitation in the NIR spectral range is preferable, as AF signal becomes weak, yet fluorescence emission still presents to some extent [17,18]. On the other hand, red/NIR AF in several cases might be useful for diagnostic purposes.

Several works reported that red/NIR AF can be used to discriminate between normal and cancer tissues in bladder, colon and breast [19,20,21]. Red AF was used to characterize biological aspects of the senescence of *C. elegans* nematodes and was shown to be a predictor of its lifespan [22]. In the work of Pansieri et al. [23]. NIR AF originated from proteins aggregation into amyloid fibrils has been described, and the origin of fluorescence signal was attributed to the supramolecular structure of protein aggregates. Htun et al. [24] made use of NIR AF to detect high-risk atherosclerotic plaques, and the source of the signal was ascribed to heme degradation products. Enhanced NIR AF signal has also found application in fluorescence-guided surgery to detect parathyroid glands [25]. Although this approach is already accepted by clinicians, the origin of fluorophore is unknown [26]. Overall, the origin of fluorophores responsible for emission in the red and NIR spectral regions is debatable.

In this paper, we aim at discussing the hypothesis about the role of oxidation in red/NIR fluorescence formation. The main idea is that formation of oxidized species may result in emergence of long wavelength absorption, which enables fluorescence excitation in a broad spectral range. This process is somewhat similar to formation of lipofuscin, the so-called aging pigment, which accumulates in cells as a result of oxidation and aggregation of proteins and lipids, and is known for its strong fluorescence [27,28]. Lipofuscin granules are brightly fluorescent and can be excited in the yellow and red spectral regions, and fluorescence of lipofuscin is extensively studied for retina [29,30], liver and kidneys [11]. It is also known that direct oxidation may induce formation of novel fluorescence bands [31], for instance, in the case of aromatic acid containing proteins.

Here we aim at demonstrating that oxidation of organic compounds naturally presented in biological tissues may result in an increase in fluorescence emission with a broad excitation spectrum, including excitation in the red region. First, we demonstrate fluorescence enhancement for a number of model solutions, exposed to photooxidation. Second, we show how an induction of oxidation processes in cells may result in emergence of red fluorescence from oxidation products. For this, we assess keratinocytes AF properties after UV irradiation, which is known to be an appropriate model of cells’ oxidative stress [32].

## 2. Results

### 2.1. Photooxidation-induced Emergence of Broadband Excitation Spectrum in Model Solutions

To demonstrate that oxidation of biomolecules may result in long wavelength absorption and NIR fluorescence formation, aqueous solutions of tryptophan (Trp) and DNA were exposed to UV irradiation.

**Figure 1:**
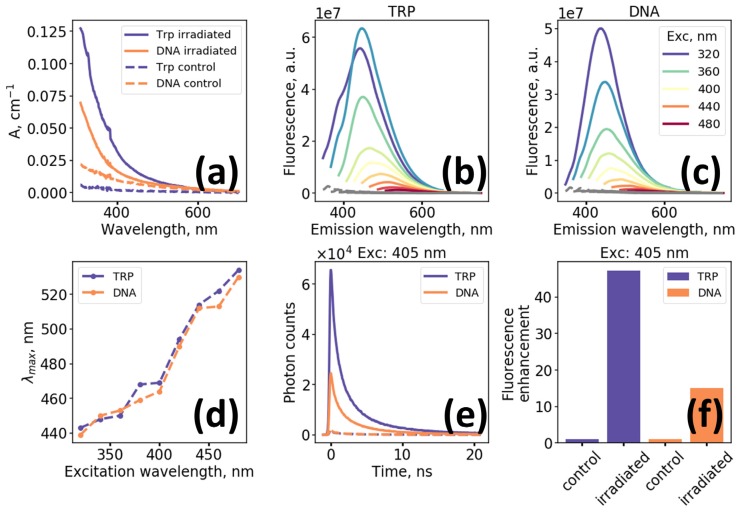
Photooxidation-induced changes of optical properties of tryptophan (Trp) and DNA water solutions (**a**) Absorption spectra of irradiated and non-irradiated with UV solutions of Trp and DNA; (**b**) Fluorescence spectra of UV-irradiated Trp (0.05 mg/mL) solution; (**c**) Fluorescence spectra of UV-irradiated DNA (1 µg/µL) solution; (**d**) The dependence of the wavelength of maximum of the fluorescence spectrum on the excitation wavelength for UV-irradiated Trp and DNA solutions; (**e**) Fluorescence decay curves of irradiated (solid curves) and non-irradiated (dashed curves) Trp and DNA solutions obtained at 405 nm excitation; (**f**) Fluorescence enhancement factors for irradiated Trp and DNA solutions obtained at 405 nm excitation. UV irradiation of the Trp and DNA samples was performed at λ = 254 nm with intensity 10 mW/cm^2^ during 3 h (~100 J/cm^2^) at 20 °C.

Figure 1a demonstrates evolution of the absorption spectra of Trp and DNA solutions after UV irradiation (λ = 254 nm at 10 mW/cm^2^ intensity during 3 h (~100 J/cm^2^) at 20 °C). Initial spectra are characterized by the presence of absorption band in the UV and negligible absorption in the range above 400 nm. Following irradiation, broadband structureless absorption emerges, which monotonically decreases with wavelength, also resulting in a distinguishable yellowing of the solutions. Of interest is the emergence of long wavelength (>600 nm) absorption, which is a necessary prerequisite for red and NIR fluorescence excitation. Figure 1b,c present fluorescence spectra of irradiated Trp and DNA solutions measured for excitation wavelengths varied from 320 to 480 nm where non-irradiated compounds do not exhibit intense fluorescence signal. We would like to highlight the monotonic decrease of fluorescence intensity and increase of the position of maximum in the fluorescence emission spectrum with the increase in excitation wavelength which is demonstrated in Figure 1b–d. 

Both photo-oxidized Trp and DNA solutions demonstrate multiexponential kinetics (Figure 1e) of fluorescence decay excited at 405 nm. Using biexponential fitting procedure with deconvolution we obtained that mean fluorescence decay lifetime of both samples was of the order of ~ 2.2 ns, while the enhancement of fluorescence excited at 405 nm, calculated as an integral over fluorescence decay curves, was ~ 15 for irradiated DNA and ~ 45 for irradiated Trp solutions (Figure 1f). The effects of UV-irradiation on the changes of optical properties of the model solutions in buffer (PBS) were similar to that observed for aqueous solutions (Figure S1).

We also checked the possibility of detection of the red edge excited fluorescence from oxidation products of aromatic amino acids. To obtain intense signal we irradiated the water solutions of Trp and tyrosine (Tyr) with concentration of 1 mg/mL and 0.2 mg/mL, respectively, with UV light (λ = 254 nm) at 10 mW/cm^2^ intensity during 6 h (~200 J/cm^2^) at 20 °C and then dried the solutions on the standard glass slides. The obtained powders were then measured using fluorescence lifetime imaging (FLIM) technique with 640 nm picosecond pulsed excitation and emission detection in the 660–720 nm range.

**Figure 2:**
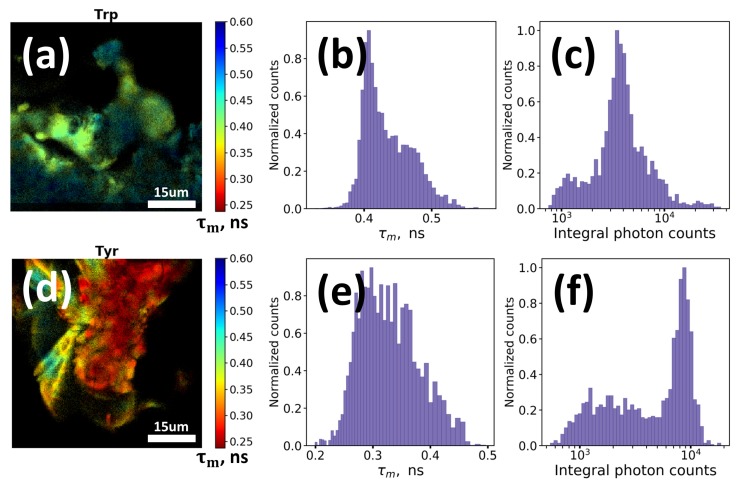
Fluorescence lifetime images, mean fluorescence lifetime 𝜏_m_ distribution and fluorescence intensity distribution obtained from dried samples of UV-irradiated tryptophan (**a**–**c**) and tyrosine (**d**–**f**). The measurements were performed at 640 nm excitation and detection in 660–720 nm spectral region. UV irradiation of the tryptophan and tyrosine solutions (1 mg/mL) was performed at λ = 254 nm with intensity 10 mW/cm^2^ during 6 h (~200 J/cm^2^) at 20 °C.

Intense AF signals from UV irradiation-induced oxidation products of both Trp (Figure 2a–c) and Tyr (Figure 2d–f) were observed while in case of non-irradiated samples the signal was very weak and almost non-detectable. Figure 2b,e demonstrate mean lifetime and Figure 2c,f present integral intensity of the observed fluorescence. Mean fluorescence lifetime 𝜏*_m_* appeared to be short (~0.3–0.5 ns) and heterogeneously distributed over different regions of the imaged powders (corresponding FLIM images are demonstrated in Figure 2a for Trp and Figure 2d for Tyr), that can be presumably explained by heterogeneous composition of the Trp and Tyr oxidation products.

Thus, as it can be seen, oxidation of aromatic amino acids and DNA may result in formation of long wavelength (λ ≥ 400 nm) absorption and corresponding red fluorescence. Even at rather low concentrations of monomeric precursors (~1 mg/mL) long wavelength fluorescence signal from oxidation products can be easily detected (Figure 2). Concentrations of amino acids, lipids, proteins and other constituents in cells and tissues are several orders higher than in model solutions we used in the experiments. Hence, it can be assumed that in case of cells and biological tissues fluorescence of oxidation products may provide for a certain contribution to the resulting fluorescence signal, especially in the red region of spectra, where the impact of other endogenous fluorophores is low. To verify this hypothesis we conducted a series of experiments on AF measurements in human keratinocytes under oxidative stress induced by UV irradiation and hydrogen peroxide (H_2_O_2_).

### 2.2. Alterations of AF in Cells after Exposure to UV Revealed by Flow Cytometry

We made use of flow cytometry with multispectral excitation (405, 488 and 638 nm) and emission detection (from 450 up to 700 nm) to assess changes of AF properties of human skin keratinocytes following exposure to UV radiation. Irradiation of the HaCaT keratinocytes cell line was induced by UV source (λ = 254 nm), and the doses of radiation varied from 25 up to 200 mJ/cm^2^. No changes were observed immediately after irradiation, suggesting the absence of significant amount of directly oxidized species (Figure S2). Pronounced fluorescence enhancement and changes in the scattering signals occurred several hours later.

Five hours after irradiation, median forward scattering (FSC) values decreased by ~15% (for 25 and 50 mJ/cm^2^ doses) and by 50% (200 mJ/cm^2^ dose) compared to the control sample (Figure S3). After 15 h after irradiation, a decrease in the median FSC value by ~ 40–50% was observed for all irradiation doses. Changes of FSC values were found to be statistically significant for all tested subgroups at a significance level of *p <* 10^−4^ while changes in side scattering (SSC) values for some doses were less observable (Figure S5a,b).

When analyzing changes of fluorescence, we additionally normalized the AF values to the FSC value for each cell to account for alteration in cell’s size; similar procedure was used in [33].

**Figure 3:**
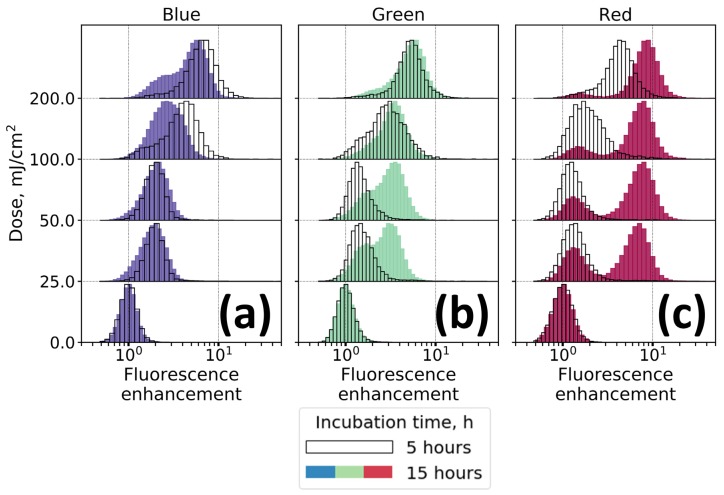
Distribution of fluorescence enhancement factors for UV-irradiated (λ = 254, 10 mW/cm^2^) keratinocytes in different spectral channels: (**a**) Blue (ex = 405/em = 450 (45) nm); (**b**) Green (ex = 488/em = 525 (40) nm); (**c**) Red (ex = 638/em = 660 (10) nm). Black and white histograms correspond to 5 h of incubation after irradiation and colored histograms correspond to 15 h of incubation under standard conditions.

AF was analyzed at three excitations (405, 488 and 638 nm). Hereinafter, we will focus on three emission channels, namely, FL Blue (ex = 405/em = 450 (45) nm), FL Green (ex = 488/em = 525 (40) nm) and FL Red (ex = 638/em = 660 (10) nm). Figure 3 presents the distribution of fluorescence enhancement factors (EF) in irradiated cells, calculated as the fluorescence intensity normalized to the corresponding median value of the fluorescence signal in the control sample of intact non-irradiated cells. The changes in the values of fluorescence enhancement for all subgroups of cells irradiated with doses of 25, 50, 100 and 200 mJ/cm^2^ are statistically significant (Kruskal-Wallis test) with the significance level of *p <* 10^−4^ (Figure S5c–e).

Several facts associated with changes of keratinocytes AF after UV irradiation should be noted. First, in the Blue channel within 5 h after irradiation an increase in the AF was proportional to the radiation dose (Figure 3a and Figure S4a), while no further fluorescence enhancement in the Blue channel was observed 15 h after irradiation.

For the Green channel, the distribution of fluorescence signal values of cells irradiated with a dose of 25 and 50 mJ/cm^2^ remained almost unchanged within 5 h after irradiation (the median fluorescence enhancement factor was lower than 2), while for the doses of 100 and 200 mJ/cm^2^ for the same period of time the median enhancement factor (EF) was ~3.5 and 5, respectively (Figure 3b). Unlike the fluorescence signal in the Blue channel, for low irradiation doses AF enhancement in the Green channel was observed within 15 h after irradiation (Figure 3b).

For the Red channel, this trend was even more pronounced (Figure 3c). The distribution of EF in the Red channel was bimodal, indicating the presence of cells without signal enhancement at any irradiation dose. Five hours after irradiation, a significant increase in fluorescence (with a median EF of ~ 4.5) was observed only for the sample with the irradiation dose of 200 mJ/cm^2^. However, after 15 h of incubation, the fluorescence signal increased more than a factor of 5 for all radiation doses.

We emphasize that the observed changes in the AF values are not associated with a change in the size of the cells, since the median forward scattering values changed by 50% maximum, while the values of the AF increased much more significantly (histograms of fluorescence values not normalized to FSC are shown in Figure S6). Also importantly, the level of fluorescence enhancement in cell debris was significantly lower than for the cells (Figure S7), suggesting that AF increase is caused by biochemical processes within cells rather than by direct influence of UV radiation. Similar effect, i.e., fluorescence enhancement in the visible range, was observed when using another oxidative stress inducer, namely, hydrogen peroxide H_2_O_2_ (Figure S8).

Asynchronous behavior of AF excited at different wavelengths suggests the formation of a complex system of fluorophores induced by UV irradiation. Indeed, in the case of formation of a single type of fluorophore responsible for fluorescence enhancement, one would observe a synchronous change in the AF with time. Moreover, the possibility of detecting AF in the Red channel seems intriguing, since red AF is a rather specific marker, and a small number of endogenous fluorophores that are excited in this spectral range are known. As it has been demonstrated in the previous section, such alterations of optical properties may occur during nonspecific photooxidation of the organic components of the cell (amino acids, lipids, proteins), which results in a generation of long wavelength absorption tail and, therefore, in a possibility of AF excitation in the red region of spectrum. As UV irradiation is a well-known inductor of oxidative stress in cells, it could be assumed that the mechanism of fluorescence enhancement observed in our experiments is related to oxidative processes triggered by absorption of UV photons by cell compartments.

### 2.3. UV-irradiation Induced Changes in AF of Keratinocytes: FLIM and Confocal Microscopy Results

FLIM technique was used to investigate changes of fluorescence properties of cells induced by UV irradiation. FLIM measurements were performed for non-irradiated HaCaT cells (control) and HaCaT cells irradiated with UV light (λ = 254 nm, intensity 10 mW/cm^2^) at 100 mJ/cm^2^ dose and incubated afterwards for 15 h under standard conditions. Two excitation wavelengths (402 nm and 640 nm) were used.

**Figure 4:**
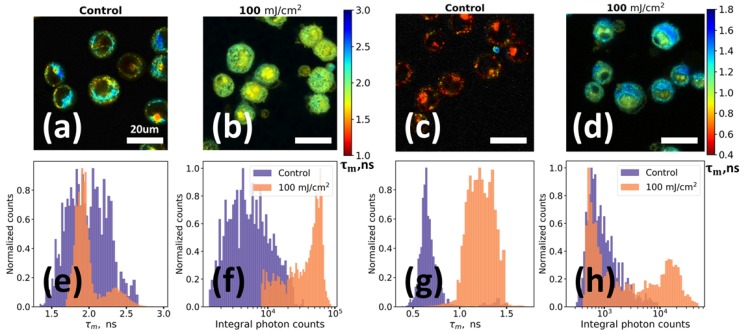
UV irradiation-induced changes of HaCaT keratinocytes autofluorescence revealed by FLIM: (**a**,**c**) Fluorescence lifetime images of intact non-irradiated (control) keratinocytes upon excitation at 402 nm (**a**) and 640 nm (**c**); (**b**,**d**) Fluorescence lifetime images of UV-irradiated keratinocytes upon excitation at 402 nm (**b**) and 640 nm (**d**); (**e**,**g**) Histograms of mean lifetime for control and irradiated keratinocytes for excitation at 402 nm (**e**) and 640 nm (**g**); (**f**,**h**) Histograms of integral fluorescence photon counts for control and irradiated keratinocytes for excitation at 402 nm (**f**) and 640 nm (**h**). (**a**–**d**) Images are color-coded by mean fluorescence lifetime (τ_m_) and additionally contrasted for clear visibility. Histograms presented in Figure (**e**–**h**) were calculated only in the regions of cells excluding values from the background. UV irradiation was performed at λ = 254 nm with a dose of 100 mJ/cm^2^. After that cells were incubated during 15 h under standard conditions.

Figure 4a–d demonstrate mean fluorescence lifetime 𝜏_m_ spatial distribution within the control non-irradiated and UV irradiated keratinocytes. Detailed information about changes in fluorophores’ parameters can be obtained from the distributions of mean fluorescence lifetime (Figure 4e,g) and integral photon counts (Figure 4f,h) for the studied samples. Two main facts can be observed: (i) while the nuclei region is dark at 402 nm excitation in the control sample, it is fluorescent in UV irradiated cells; and (ii) while cells from the control sample are almost non-fluorescent at 640 nm excitation, pronounced red emission is observed for the UV irradiated cells. The first fact is in agreement with both flow cytometry (Figure 3a) and confocal microscopy (Figure S9) data, and the second fact is in agreement with the flow cytometry data (Figure 3c).

For fluorescence excited at 402 nm a rather high fluorescence intensity of the cells both in control and UV irradiated samples was observed. In the control sample, the fluorescence signal was mainly located in the region of the cytoplasm, where at this excitation wavelength NAD(P)H and flavins can be readily excited [4,34], while nuclei had a negligible fluorescence signal (Figure 4a). Irradiated sample demonstrated radical changes of fluorescence signal. UV exposure of cells led to an increase in integrated fluorescence intensity (EF ~ 8), changes of the mean fluorescence lifetime (Figure 4e) and in spatial localization of the fluorophores (Figure 4f). For instance, in the Figure 4b one may see that an intense fluorescence is observed from the region of cells’ nuclei with a slightly shorter decay time compared to the cytoplasm of irradiated cells (1.7 ns vs. 2.2 ns, Figure 4e).

The alterations of the fluorescence signal in the UV irradiated sample were even more dramatic for the red (640 nm) excitation. From the control sample only a weak signal with very short decay lifetime comparable to that of instrument response function (IRF) was observed (Figure 4c; note that the image is contrasted for clear visibility of cells). We suppose that the main source of this signal in the sample of non-irradiated keratinocytes is the elastic scattering on the cells’ fragments that erroneously passes through optical band pass filters. However, in contrast to the control, in the irradiated sample (Figure 4d), an intense (at least an order of magnitude higher) fluorescence signal was observed (Figure 4h). As in the case of excitation at 402 nm, the red fluorescence signal in the irradiated sample was localized both in the cytoplasm and in the region of the cells’ nuclei, while the characteristic decay times of fluorescence in the nuclei were shorter than in the cytoplasm (0.9 ns vs. 1.2 ns, Figure 4g). Discrepancies in the lifetimes, obtained at different excitation wavelengths, are probably connected with the complex heterogeneous structure of oxidation products and their photophysical properties.

We studied the changes of HaCaT keratinocytes morphology following UV irradiation at high dose (~100 mJ/cm^2^) using confocal microscopy technique. The results are presented in Supplementary Information in Figures S10–S12. The bright field images of the control intact and irradiated keratinocytes after 5 h incubation under standard conditions are shown in the Figure S10. UV irradiation led to significant morphological changes of the cells (Figures S11 and S12). Namely, cells shrinkage with a clearly pronounced reduction of cytoplasm volume (Figure S12a) and membrane blebbing around the affected cells (Figure S12b) were observed. The whole sample contained a lot of small debris which looked like defragmented residues of cells. Such behavior of the cell culture can be related to the last stages of apoptotic death, which could be expected at such a high UV dose.

In agreement with the flow cytometry data, an increase in AF was observed in UV irradiated cells measured 5 h after UV-irradiation (~100 mJ/cm^2^) using confocal fluorescence microscopy. The most dramatic increase in AF was observed in the Blue channel (ex = 405/em = 450 (45) nm), where the EF reached 3–5 (Figure S9). The analysis of AF distribution within the cell monolayer showed that fluorescence increased both in cytoplasm and nuclear region cells. The most pronounced fluorescence enhancement was observed in the blue channel, as longer incubation is required for significant alteration of green and red fluorescence (Figure 3c).

To study the fate of irradiated cells we performed cytometric apoptosis assay using Annexin V/propidium iodide staining. The results are presented in the Figure S13 and clearly demonstrate that in each irradiated sample there are fractions of apoptotic and necrotic cells. When the cell sample is irradiated with UV doses 20–100 mJ/cm^2^ the apoptotic fraction is dominating.

In order to estimate the oxidation process within living cell under UV irradiation we performed a Singlet Oxygen Sensor Green (SOSG) probing test using confocal microscopy (the images are presented in Figure S14). It can be seen that at high dose of UV (100 mJ/cm^2^) reactive oxygen species (ROS), including singlet oxygen, appear in all compartments of the cell: both in nucleus and non-nucleus regions.

## 3. Discussion

The major aim of the present work was to illustrate the principal possibility of long wavelength absorption and red fluorescence formation in model solutions and cells in vitro as a result of oxidation processes. In case of solutions of aromatic amino acid Trp and DNA exposed to UV radiation at the absorption maxima of these molecules, photooxidation takes place [35,36], which is known to be capable of generating chemical species with different absorption and fluorescence spectra. For instance, due to its low ionization potential Trp can be readily photo-oxidized [37]. This leads to the formation of various oxidation products, some of which (e.g., kynurenine) are fluorescent in the blue-green spectral region and appear both in solutions of Trp [38] and in proteins [39,40]. In our experiments broad structureless absorption and excitation-dependent fluorescence appeared upon UV exposure of Trp and DNA (Figure 1a–c). Similar behavior of optical properties has been described for a range of systems, which exhibit novel intrinsic fluorescence upon self-assembly [40,41]. The observed monotonic dependence of maximum in the fluorescence spectrum on excitation wavelength (Figure 1d) is known as the red edge excitation effect and has been described for heterogeneous systems of fluorophores as graphene oxide, organic quantum dots, aggregates of small molecules, etc. [42].

On the one hand, the apparent visible fluorescence can be explained solely by the fact that as a result of oxidation a large number of different molecular species appear, including fluorescent ones, and the overall optical response of the system is due to superposition of signals from individual molecules. On the other hand, optical properties of the whole system can originate from intermolecular interaction between the newly formed molecular species. Both hypotheses are considered in the literature for the systems, which exhibit optical properties similar to that of UV-irradiated Trp and DNA —carbon and graphene quantum dots, where excitation-dependent fluorescence is explained both by size-dependent effects, the occurrence of surface states near oxidized groups, and ultra-slow relaxation of the solvent [42]. Emergence of visible AF with similar properties has been also reported for amyloid fibrils: in [23] aggregation of proteins into fibrillar structures was hypothesized to be main source of visible and NIR fluorescence. In [43] a proton transfer mechanism was considered as a molecular basis for the emerging visible fluorescence in fibrils. In [31] either aggregation-induced or oxidation hypotheses were considered to explain emerging so-called deep blue AF of amyloid fibrils. “Non-traditional intrinsic luminescence” observed in supramolecular self-assemblies lacking traditional luminophores was explained as the result of clustering and physicochemical confinement of normally non-emissive, electron rich, hetero-atomic and functionalized moieties [44].

The hypothesis tested in this work implies that oxidation induced by biochemical processes may also result in the formation of red edge excitation similarly to the case of direct photooxidation. We observed that a substantial increase in AF in cells appeared only several hours after irradiation (Figure 3) and was not present in cells’ debris (Figure S7). These facts indicate that oxidation products in cells are formed as a result of intracellular biochemical processes but not under the direct photooxidation. Biochemical status of cells may change due to the photochemical reactions induced by UV light or through ROS generation. UV irradiation may excite DNA molecules leading to the formation of photolesions, in particular cyclobutane pyrimidine dimers (CPD) [45,46]. The CPD photoproducts interfere with base pairing during DNA replication, leading to signature mutations within genes sequences regulating cell cycle and genetic material reparations resulting in disabling of antioxidant cells protection [47]. Absorption of UV radiation by aromatic-rich molecules may also result in generation of ROS in cells which may damage DNA and structural proteins, e.g., keratin, which is known to exhibit enhanced fluorescence upon oxidation [48] and initiate apoptotic pathways [49]. We observed the generation of ROS within keratinocytes under UV irradiation by visualization with SOSG staining (Figure S14).

Changes of AF signal in eukaryotic cells were previously reported to be due to the activation of molecular pathways aimed at dealing with the life-threatening processes. An exposure to different stressors led to a strong increase in the AF of *E. coli*, yeast cells and human cell lines [33]. For instance, exposition of different strains of *E. coli* to ampicillin induced an increase in green AF (ex = 488 nm/em = 530). The authors suggested flavins to be the major source of AF increase and demonstrated that the excitation spectrum of cells corresponds to that of flavins. Increased AF was observed in the human and murine cells following ionizing radiation [50]. Using flow cytometry with a 488 nm laser excitation and AF signal detection in the 515–545 nm range it was demonstrated that mean fluorescence intensity of cells after X-ray irradiation increases in a dose- and time dependent manner. The authors attributed this effect to an increase in flavin adenine dinucleotide (FAD) concentration and demonstrated increased expression of genes encoding diverse flavoproteins after irradiation, which are involved in energy production and ROS quenching. Measurements of AF assessed by means of the confocal microscopy in a wide spectral range were used to characterize biological aspects of the senescence of *C. elegans* nematodes [22]. The authors report that blue and green AF increase very little across aging except for a peak near death, while red AF increases linearly over time. However, the origin of fluorophore responsible for red emission was not studied. In [51] the authors propose the usage of green endogenous AF as a real-time quantification of cellular senescence in human mesenchymal stromal cells based on label-free flow cytometry analysis and the involvement of lipofuscin in the AF increase was discussed. In our previous work it was shown that the appearance of the fluorescence signal in the living cells can be governed by the formation of photoproducts: for instance, the irradiation of erythrocytes with 760 nm femtosecond laser pulses led to the formation of hemoglobin photoproducts which were characterized by a broad excitation spectrum [52].

In this work, we demonstrated that UV irradiation led to dramatic enhancement of fluorescence in cells, which exhibited broad excitation spectrum covering from blue to red and was different from that of flavins. It was observed that nuclei, which exhibit low AF in control samples, become brightly fluorescent after UV irradiation. The broad spectrum of fluorescence excitation and localization of emission within cells suggests that the observed effect could be due to the formation of oxidation products, as observed in model solutions. The dramatically elevated level of ROS in keratinocytes after UV exposure was additionally demonstrated by using the SOSG assay (Figure S14). Overall, the obtained results favor the role of oxidation in the formation of red edge fluorescence in cells.

One more important conclusion can be made from the presented data. Namely, dramatic enhancement of fluorescence at blue excitation indicates that the corresponding signal cannot be attributed solely to NADH and FAD. Hence, the presence of other fluorophores, in our case, originated from oxidation of cells components (photoproducts), may interfere the results of metabolic imaging when using the standard concept for data interpretation. Additionally, the effect of fluorescence photobleaching [53] can also influence the results interpretation and should be investigated in detail in relation to the fluorescence-active chromophores.

## 4. Materials and Methods

### 4.1. Photooxidation of Model Solutions

Aqueous solutions of L-Tryptophan (Trp, Sigma Aldrich, Darmstadt, Germany) and DNA were used as model systems to demonstrate that UV irradiation-induced oxidation of biomolecules may result in long wavelength absorption and NIR fluorescence formation. Concentration of the prepared stock solution of Trp was 0.05 mg/mL (~10^−4^ M). The model DNA sample was the control plasmid DNA (7500 bp) isolated from *E. coli* organisms with QIAprep Spin Miniprep Kit (Qiagen, Germantown, MD, USA) according to the manufacturers’ protocol. The quality and quantity of the DNA sample was estimated with NanoPhotometer NP80 (Implen, München, Germany). Concentration of DNA in the sample was 1 µg/µL. All samples were photo-oxidized by irradiation with a UV-lamp (λ = 254 nm, intensity 10 mW/cm^2^) for 3 h (100 J/cm^2^) at 20 °C.

For fluorescence lifetime imaging experiments, aqueous solutions of Trp and L-tyrosine (Tyr, AppliChem, Darmstadt, Germany) were prepared. Concentrations of the stock solutions were 1 mg/mL and 0.2 mg/mL for Trp and Tyr, respectively. Stock solutions with volume of 2 mL were photo-oxidized by irradiation with a UV-lamp (λ = 254 nm, intensity 10 mW/cm^2^) for 6 h (200 J/cm^2^) at ambient temperature (25 ± 2 °C). After irradiation, samples were dried on a slide glass for 3 h at 40 °C.

### 4.2. Cells Culture Conditions and Growth Media

Human keratinocytes cell line HaCaT was used in the present work. The cells were kindly presented by laboratory of cellular biology of N.K. Koltzov Institute of Developmental Biology of Russian Academy of Sciences (Moscow, Russia). Cells were cultured in 25 cm^2^ tissue culture plastic flasks with a filter cap (SPL Lifesciences, Pocheon, Korea) under standard conditions (humidified atmosphere of 5% CO_2_ at 37 °C) using Dulbecco’s Modified Eagle’s medium (DMEM) (PanEco Ltd., Moscow, Russia) supplemented with 10% of fetal bovine serum and 0.32 mg/mL L-glutamine (PanEco Ltd.). Cells passaging was performed three times a week by incubation of cells in vapors of 0.25% Trypsin-EDTA in Hank’s balanced salt solution (PanEco Ltd.) during 5 min at 37 °C with preliminary rinsing the cells layer with warm Versene solution (PanEco Ltd.). Twenty four h prior to the UV irradiation HaCaT cells were placed in 35 mm diameter Petri dish in concentration 75000–100000 cells per mL. During 24 h of incubation all cells dropped down to the bottom of the dish and formed a monolayer.

### 4.3. UV Irradiation Protocol for the Cells Culture

UV irradiation of the cells was performed in the Petri dish using a UV-lamp (λ = 254 nm, intensity 10 mW/cm^2^). The irradiation dose was adjusted by the irradiation duration and the thickness of DMEM medium above the cells layer. The DMEM medium in which cells were irradiated was changed with the fresh one after the UV exposure. The detailed conditions of keratinocytes UV irradiation for each dose are provided in the Supplementary Table. After the UV exposure, the cells were incubated under standard conditions during 5 or 15 h. Then the incubation medium was removed and cells were gently detached from the dish bottom surface by 1 mL of trypsin-EDTA solution. The acquired suspension of cells was then measured using flow cytometry, FLIM and confocal microscopy.

### 4.4. Cells Staining Protocols

To characterize the oxidative stress induced by UV irradiation we used the Singlet Oxygen Sensor Green (SOSG) reagent (S36002 Thermo Fisher Molecular Probes, Eugene, OR, USA) which is widely used for the imaging of the production of singlet oxygen in living cells [54]. Cells were placed in the glass bottom Petri dish, irradiated with UV at 100 mJ/cm^2^ and incubated for 5 h under standard conditions. After the incubation both irradiated and non-irradiated intact cell samples were supplemented with 10 µL of SOSG stock solution (concentration 1.6 mM in methanol) and incubated for 30 min. Then, the medium was removed and the cells were washed 3 times in Dulbecco’s phosphate-buffered saline (DPBS, PanEco Ltd.). After that the samples were measured using the confocal microscope (Olympus FV10i, Tokyo, Japan).

To investigate the mechanisms of cells morphological alterations after UV exposure we performed staining with FITC Annexin V Apoptosis Detection Kit I (BD Pharmingen FITC Annexin Apoptosis Kit I 556547, San Jose, CA, USA), which allows to estimate the changes in membrane structural integrity at the latest stages of cell death resulting from either apoptotic or necrotic processes. The experiment involved cells samples irradiated by UV with 25, 50 and 100 mJ/cm^2^. After 15 h incubation under standard conditions the cells were stained according to the kit developer’s protocol and measured using flow cytometry.

### 4.5. Fluorescence Measurements of the Model Solutions

Steady-state fluorescence measurements were performed on FluoroMax-4 spectrofluorometer (HORIBA Jobin Yvon, Tokyo, Japan/ Longjumeau, France). Excitation-emission matrices for Trp and DNA in the 300 to 750 nm range were measured; the excitation wavelength was varied in the 280–580 nm range with 20 nm increment. The spectral width of the excitation and emission slits were set to 2 and 5 nm, respectively. Absorption spectra were measured using a UV-Vis Lambda 25 spectrophotometer (Perkin Elmer, Waltham, MA, USA) in the 200–1100 nm range.

Time-resolved fluorescence measurements of Trp and DNA solutions were performed using the time-correlated single photon counting (TCSPC) technique on the custom-built fluorometer [27]. Fluorescence was excited with a pulsed 405 nm laser diode (IOS, Saint-Petersburg, Russia) delivering 11 pJ, 40 ps FWHM pulses, driven at a repetition rate of 5 MHz. The registration system included photomultiplier (PMC-100, Becker & Hickl, Berlin, Germany) and a single photon counter module (SPC-130EM, Becker & Hickl). All measurements were carried out in a 2 mm optical path quartz cuvette at ambient temperature (25 ± 2 °C).

### 4.6. Flow Cytometry Measurements and Data Analysis

Flow cytometry experiments were performed with the CytoFLEX system (Beckman Coulter, Indianapolis, IN, USA), equipped with three excitation sources (405, 488 and 638 nm). At least 10^5^ events were detected in each experiment. Spectral channels correspond to the following excitation and emission wavelengths: Blue(ex = 405 nm/em = 450 (45) nm), Green (ex = 488 nm/em = 525 (40) nm) and Red (ex = 638 nm/em = 660 (10) nm), where for the emission channels central wavelength and (FWHM) are given. FSC and SSC channels correspond to forward and side scattering at 488 nm.

To extract signal from single cells (i.e., to exclude signal from doublets) we used the standard gating strategy, based on height and width of the forward scattering impulse (FSC-H, FSC-Width) (Figure S15). This gating strategy allowed to get rid of small cell debris and to analyze only large cells (with linear sizes differing less than ~1.5 times compared to cells in the control sample).

To verify the statistical significance of the observed differences, the pairwise comparison between flow cytometry data on the control subsample of cells and subsample, representing cells UV-irradiated with doses of 25, 50, 100 and 200 mJ/cm^2^ and incubated for 5 or 15 h, was carried out with Kruskal-Wallis H-test using Python programming language and NumPy and SciPy libraries.

### 4.7. Fluorescence Lifetime Imaging (FLIM)

FLIM measurements were performed with the Microtime 200 setup (PicoQuant, Berlin, Germany) with picosecond 402 nm and 640 nm excitation sources (40 MHz repetition rate, maximum power 50 µW, pulse duration = 40 ps). An Olympus UPlanSApo 100 × 1.4NA objective was used for capturing images with 400 × 400 pixels size with acquisition time of 0.2 ms/pixel, i.e., collection time for the whole 80 × 80 µm image was 40 s. Detection was performed in spectral channel 425–900 nm (long pass filter) for excitation at 402 nm and 660–720 nm (band pass filter) for excitation at 640 nm.

Fluorescence decay curves were acquired using the time-correlated single photon counting technique and processed using the custom-made software written in Python programming language [4]. The binning value was set to 5 (i.e., fluorescence signal was averaged over the window of 11 × 11 pixels), and only the fluorescence decay profiles with ≥20 photon counts in maximum were analyzed. Fluorescence decay curves were fitted with biexponential decay function with respect to the instrument response function (IRF) [14].

FLIM stacks were processed by custom-made Python scripts where each fluorescence decay curve was fitted by biexponential decay law with amplitudes a_1_, a_2_ and corresponding lifetimes τ_1_,τ_2_. Mean lifetime was calculated as τ_m_ = (a_1_τ_1_+ a_2_ τ_2_)/(a_1_ + a_2_).

## 5. Conclusions

In this work we evaluated and confirmed the hypothesis that heterogeneous system of oxidation products of amino acids, proteins, DNA and other cell constituents serve as the molecular source of red/NIR excited autofluorescence in biological systems. On model solutions of amino acids and DNA, we have shown that oxidation (induced by UV radiation and hydrogen peroxide H_2_O_2_) leads to the emergence of structureless absorption spectrum, which exponentially decreases with wavelength. This long wavelength absorption allows one to efficiently excite fluorescence from the products of the oxidation of amino acids and proteins in the Vis/NIR range, yet the AF properties of the emerging system of heterogeneous fluorophores still need to be carefully studied.

It was also examined whether a sufficient AF signal from oxidation products is observed during oxidative stress in biological systems. Using UV radiation as a model of oxidative stress in the HaCaT keratinocytes cell line, we discovered that oxidation products resulting from biochemical reactions in cells can make the dominant contribution to the AF signal in various spectral channels from the blue (<450 nm) to the red/infrared (>650 nm) wavelength range as it was confirmed by flow cytometry, FLIM and other techniques.

The presented results suggest the principal possibility to explain the red fluorescence excitation in aggregates of proteins and peptides, cells and tissues by the impact of oxidation products since they are inevitably presented in the tissue, and also suggest that widely excited fluorescence signal from oxidation products may lead to alteration of metabolic imaging results and has to be taken into account.

## Supplementary Materials

The following are available online, Figure S1: UV-irradiation induced photooxidation changes of optical properties of tryptophan (Trp) solution in PBS: (a)—Absorption spectra; (b)—Fluorescence spectra. The stock solution of Trp with concentration 1 mg/mL was prepared in PBS (pH 7.4, I = 0.1 M). The prepared sample in the volume of 2 mL was photo-oxidized by UV-irradiation (λ = 254 nm, intensity 10 mW/cm2) for 1 h (dose ~40 J/cm^2^) at ambient temperature 25 ± 2 °C. Figure S2: Analysis of the flow cytometry data on HaCaT keratinocytes after UV irradiation (λ = 254 nm, intensity 10 mW/cm^2^) at 100 mJ/cm^2^ dose. Measurements were performed 5 and 10 min after the UV-irradiation: (a)—blue fluorescence (FL), (b)—green FL, (c)—red FL, (d)—Forward light scattering (FSC) at 488 nm illumination. Figure S3: Analysis of flow cytometry data on HaCaT keratinocytes after UV irradiation (λ = 254 nm, intensity 10 mW/cm^2^) at different dose and different incubation time after irradiation: (a)—Forward light scattering (FSC); (b)—Side light scattering (SSC); Figure S4: Analysis of flow cytometry data on HaCaT keratinocytes after UV irradiation (λ = 254 nm, intensity 10 mW/cm^2^) at different dose and different incubation time after irradiation demonstrating increase in autofluorescence in different spectral channels: (a)—blue fluorescence (FL), (b)—green FL, (c)—red FL. Boxplots for FL enhancement with respect to FL in control sample; Figure S5: The Kruskal-Wallis test p-values of the comparison of the flow cytometry data in different detection channels: (a)—forward scattering (FSC-H); (b)—side scattering (SSC-H); (c)—fluorescence (FL) blue; (d)—FL Green; (e)—FL Red. Pairwise comparisons were made between values of the control subsample and the subsample of cells irradiated with a different dose of UV radiation and incubated for 5 (blue diagrams) and 15 (orange diagrams) hours. The dashed line indicates the level of p-value = 10^−4^. The p-value was low-limited to 10^−6^ for a better representation of plots; Figure S6: Analysis of flow cytometry data on HaCaT keratinocytes after UV irradiation (λ = 254 nm, intensity 10 mW/cm^2^) at different dose. Fluorescence (FL) intensity values in different spectral channels: (a)—Blue FL; (b)—Green FL; (c)—Red FL. Raw data without normalizing on scattering; Figure S7: Analysis of flow cytometry data on HaCaT keratinocytes after UV irradiation (λ = 254 nm, intensity 10 mW/cm^2^): Fluorescence enhancement in debris. Note that almost non fluorescence enhancement observed after UV for different incubation time after irradiation; Figure S8. Flow cytometry data (fluorescence (FL) enhancement factors and forward light scattering FSC) of HaCaT keratinocytes in different spectral channels after 19 h of cells incubation under standard conditions at the presence of peroxide H_2_O_2_ at different concentration: (a)—Blue FL; (b)—Green FL; (c)—Red FL; (d)—FSC at 488 nm illumination. The effect of H_2_O_2_ on AF of keratinocytes was studied by addition of the peroxide stock solution into the cells growth media (DMEM) to achieve desired concentration (750 and 1500 µM). After that the cells were incubated under standard conditions during 19 h. Figure S9: Autofluorescence confocal imaging of HaCaT keratinocytes monolayer (ex = 405 nm/em = 450 (50) nm): (a)—control intact sample; (b)—within 5 h after UV irradiation (λ = 254 nm, intensity 10 mW/cm^2^) at 100 mJ/cm^2^; Figure S10: Optical microscopy images of HaCaT keratinocytes: (a)—control intact non-irradiated sample; (b)—within 5 h after UV-irradiation (λ = 254 nm, intensity 10 mW/cm^2^) at 100 mJ/cm^2^. HaCaT cell line was kindly presented by laboratory of cellular biology of N.K. Koltzov Institute of Developmental Biology of Russian Academy of Sciences, Moscow, Russia; Figure S11: Optical microscopy images of HaCaT keratinocytes sample within 5 h after UV irradiation (λ = 254 nm, intensity 10 mW/cm^2^) at different doses; Figure S12: Observed morphological alterations of HaCaT keratinocytes under UV irradiation (λ = 254 nm, intensity 10 mW/cm^2^) at high dose (100 mJ/cm^2^); Figure S13: Flow cytometry data of apoptosis/necrosis processes in HaCaT keratinocytes samples after UV irradiation (λ = 254 nm, intensity 10 mW/cm^2^) at different doses: (a)—control non-irradiated cells; (b)—20 mJ/cm^2^; (c)—50 mJ/cm^2^; (d)—100 mJ/cm^2^. The assay was performed using Annexin V/propidium iodide staining. Annexin V binding was estimated in FITC (ex = 488 nm/em = 525 (40) nm) channel and propidium iodide staining was measured in PC7 (ex = 488 nm/em = 780 (60) nm) channel; Figure S14: Confocal imaging of Singlet Oxygen Sensor Green (SOSG) probe staining of HaCaT keratinocytes: (a), (c)—control non-irradiated cells; (b), (d)—within 5 h after UV irradiation (λ = 254 nm, intensity 10 mW/cm^2^) at 100 mJ/cm^2^; Figure S15: Gating strategy to determine the cluster of single cells during flow cytometry data analysis. Supplementary Table. Conditions of the UV-irradiation (λ = 254 nm) of HaCaT keratinocytes, illustration of the design of UV-irradiation and the formula of irradiation dose (D) calculations.
