# Supplementary material for: The Oxidation-Induced Autofluorescence Hypothesis: Red Edge Excitation and Implications for Metabolic Imaging
Source: Molecules. 2020 Apr 17;25(8):1863. doi: 10.3390/molecules25081863 (PMC7221974; doi:10.3390/molecules25081863)
Supplement: Supplementary file 1 [file molecules-25-01863-s001.pdf]

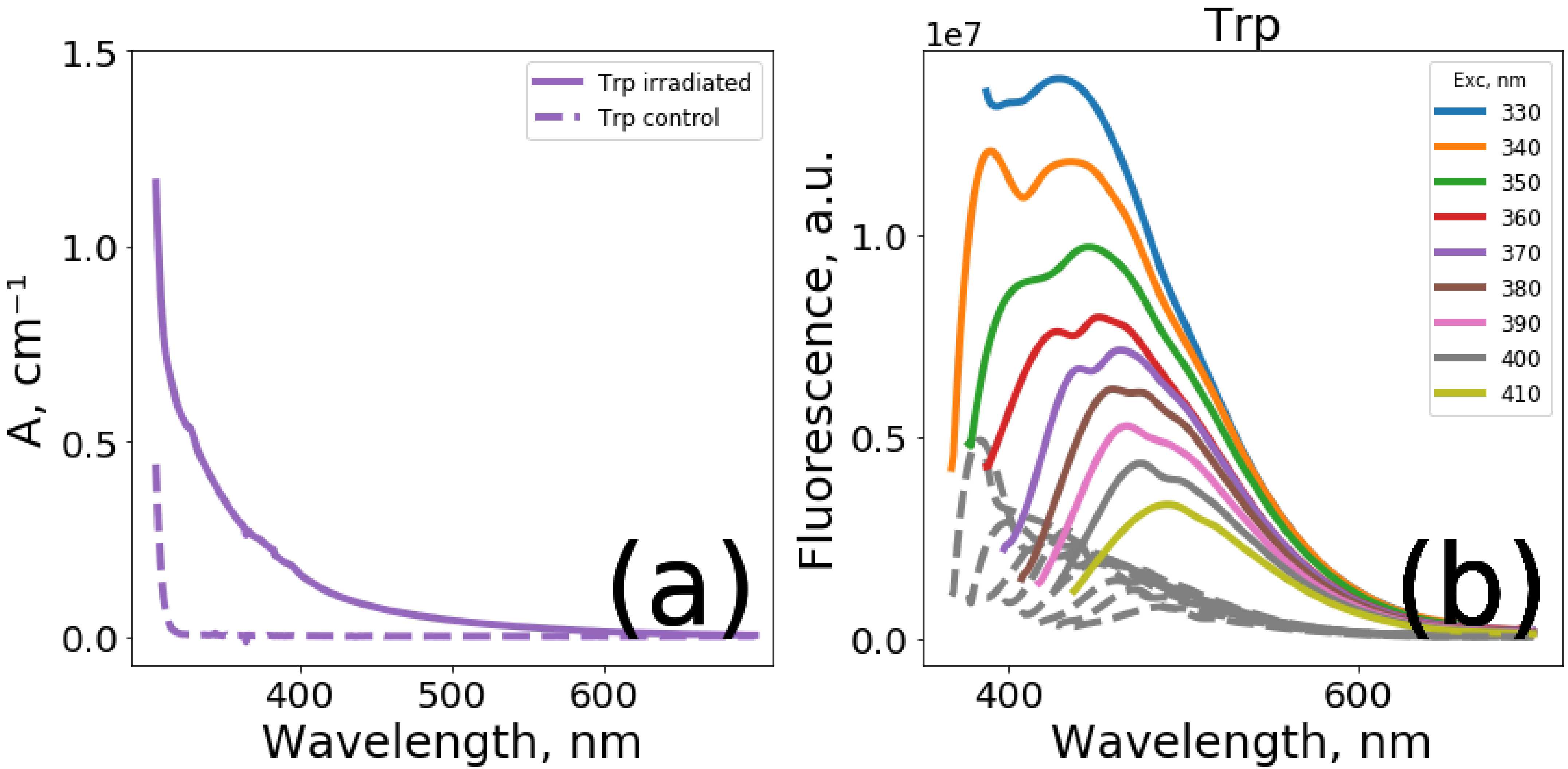

Figure S1. UV-irradiation induced photooxidation changes of optical properties of tryptophan (Trp) solution in PBS: (a) - Absorption spectra; (b) - Fluorescence spectra. The stock solution of Trp with concentration 1 mg/ml was prepared in PBS (pH 7.4, I = 0.1 M). The prepared sample in the volume of 2 ml was photo-oxidized by UV-irradiation (λ = 254 nm, intensity 10 mW/cm^2) for 1 hour (dose ~40 J/cm2) at ambient temperature 25±2°C.

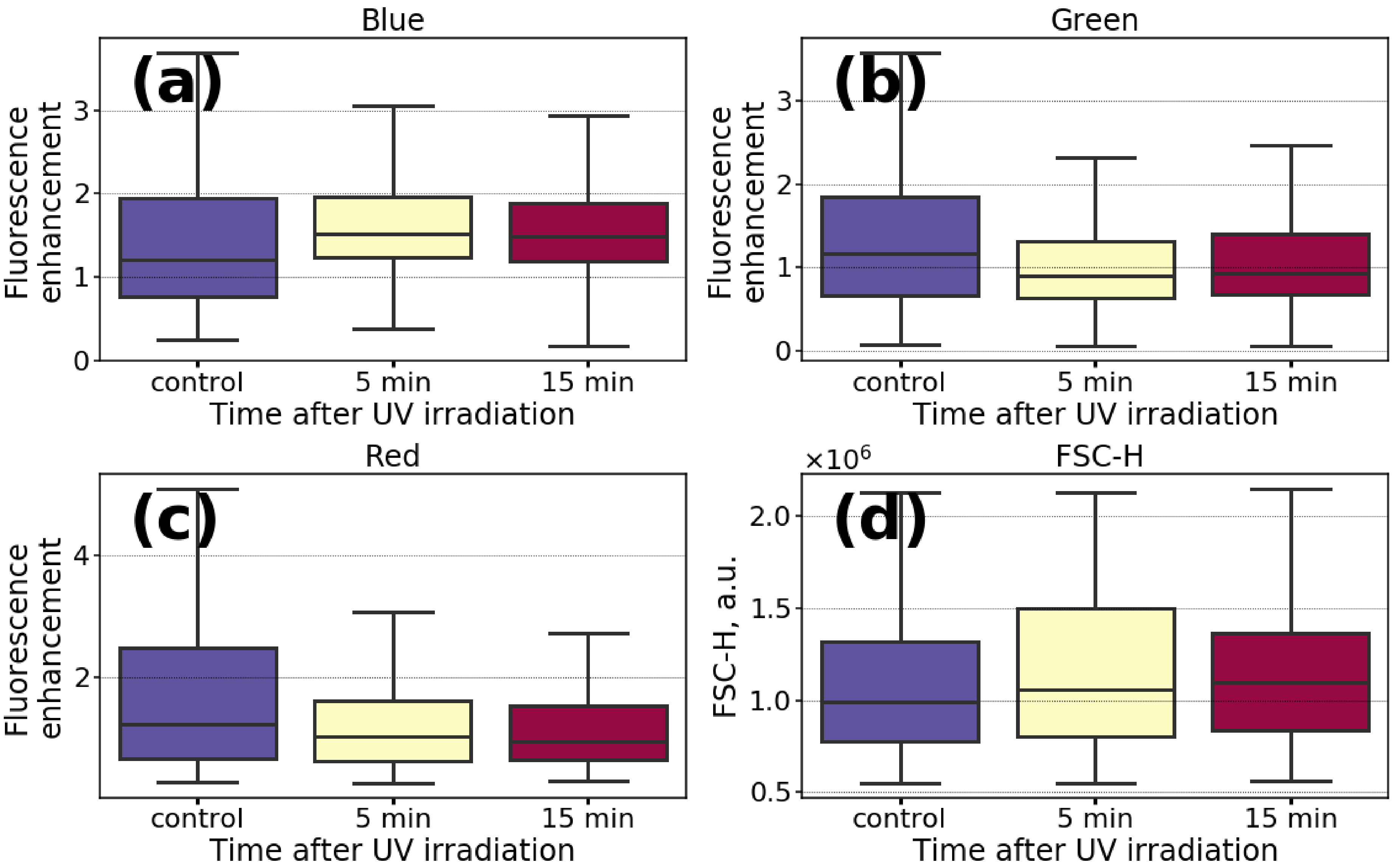

Figure S2. Analysis of the flow cytometry data on HaCaT keratinocytes after UV irradiation (λ = 254 nm, intensity 10 mW/cm^2) at 100 mJ/cm^2 dose. Measurements were performed 5 and 10 minutes after the UV-irradiation: (a) – Blue fluorescence (FL), (b) – Green FL, (c) – Red FL, (d) – Forward light scattering (FSC) at 488 nm illumination.

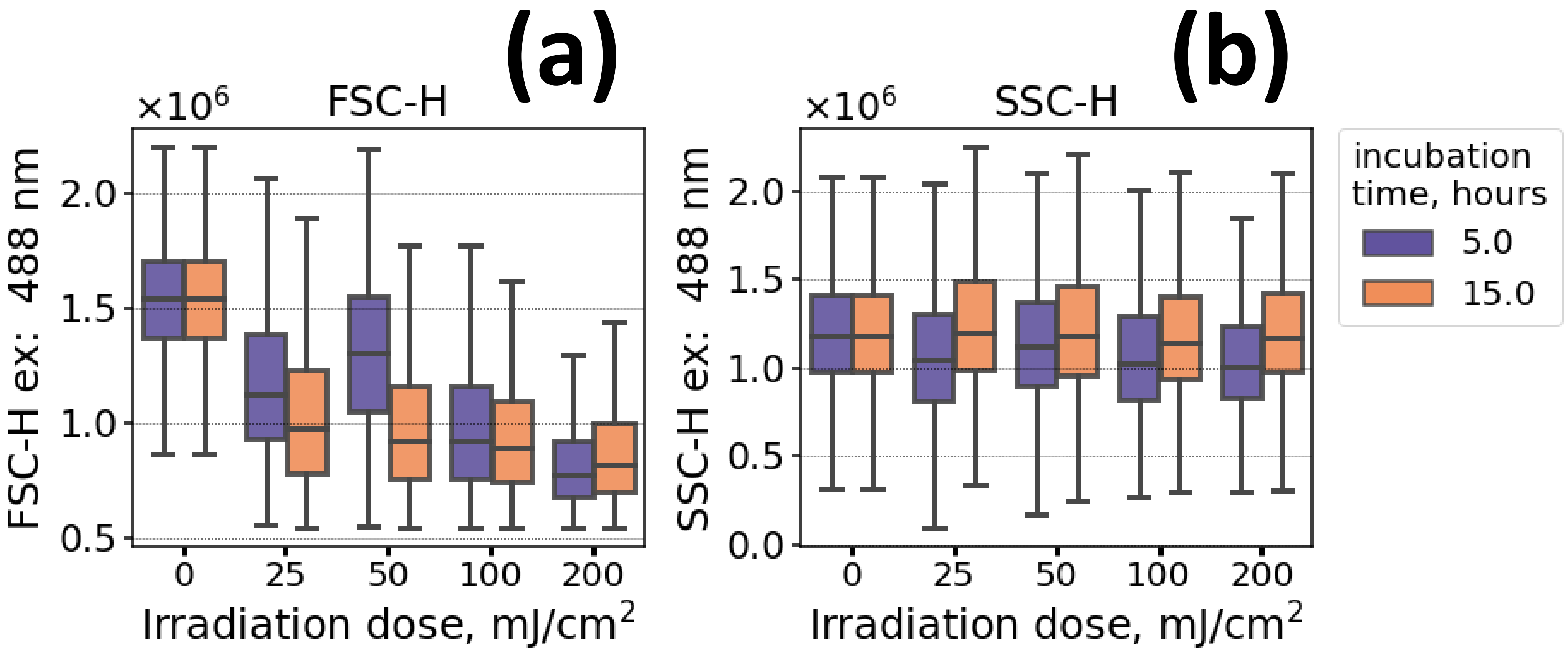

Figure S3. Analysis of flow cytometry data on HaCaT keratinocytes after UV irradiation (λ = 254 nm, intensity 10 mW/cm^2) at different dose and different incubation time after irradiation: (a) – Forward light scattering (FSC); (b) - Side light scattering (SSC).

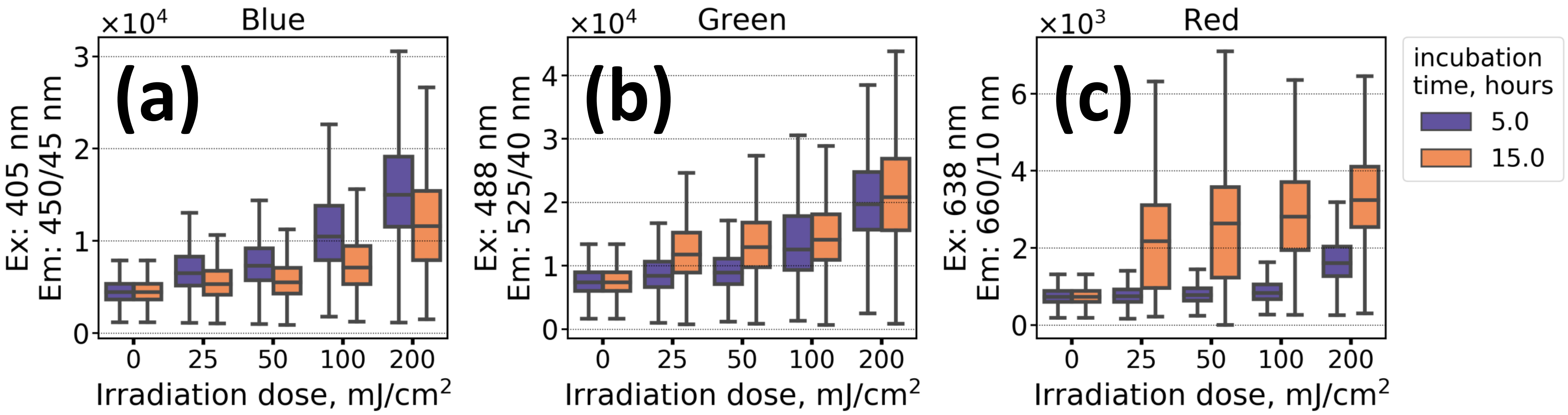

Figure S4. Analysis of flow cytometry data on HaCaT keratinocytes after UV irradiation (λ = 254 nm, intensity 10 mW/cm^2) at different dose and different incubation time after irradiation demonstrating increase in autofluorescence in different spectral channels: (a) – Blue fluorescence (FL), (b) – Green FL, (c) – Red FL. Boxplots for FL enhancement with respect to FL in control sample.

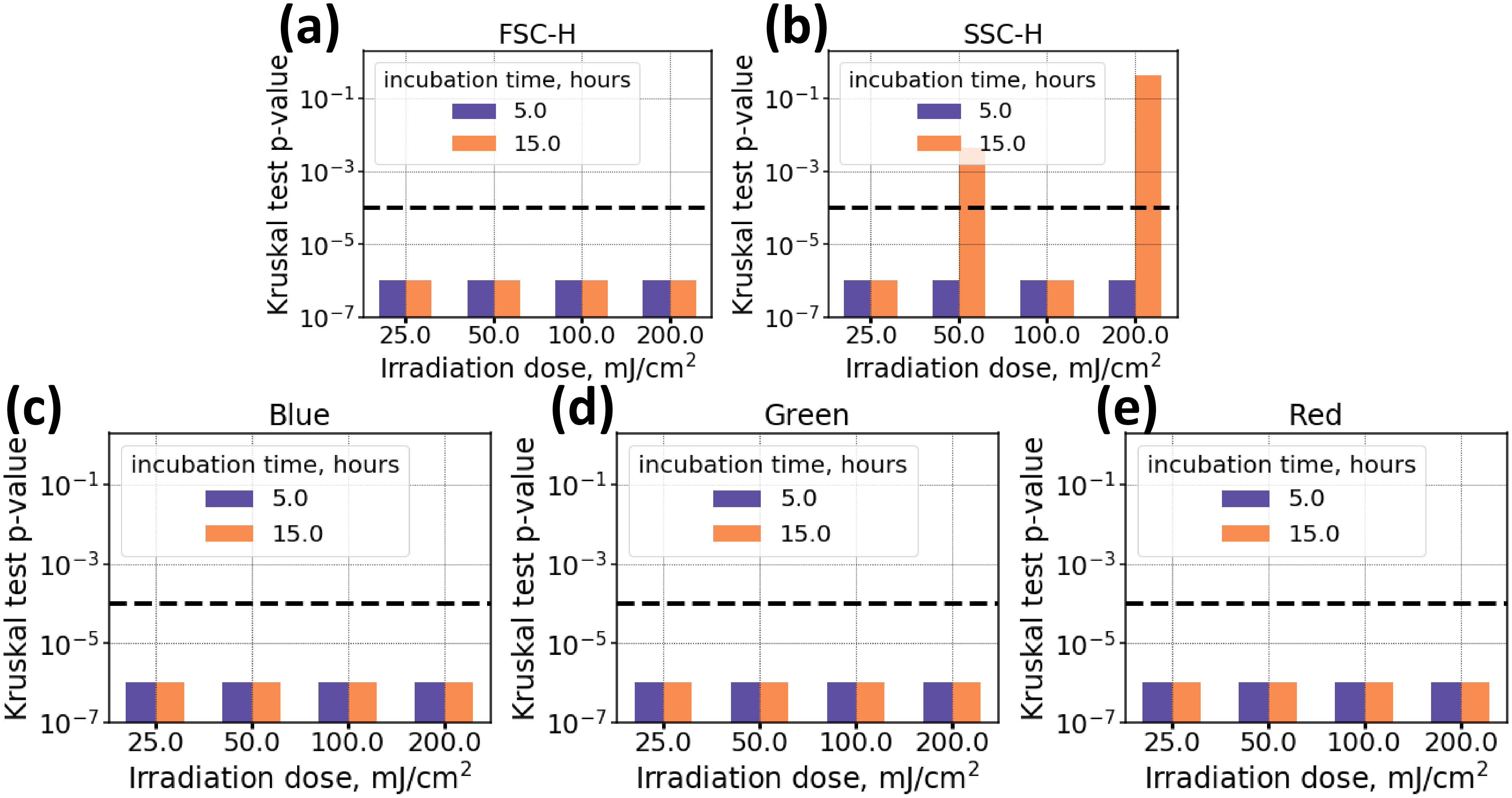

Figure S5. The Kruskal-Wallis test p-values of the comparison of the flow cytometry data in different detection channels: (a) - forward scattering (FSC-H); (b) – side scattering (SSC-H); (c) – blue fluorescence (FL); (d) - Green FL; (e) - Red FL. Pairwise comparisons were made between values of the control subsample and the subsample of cells irradiated with a different dose of UV radiation and incubated for 5 (blue diagrams) and 15 (orange diagrams) hours. The dashed line indicates the level of p-value = 10^{-4}. The p-value was low-limited to 10^{-6} for a better representation of plots.

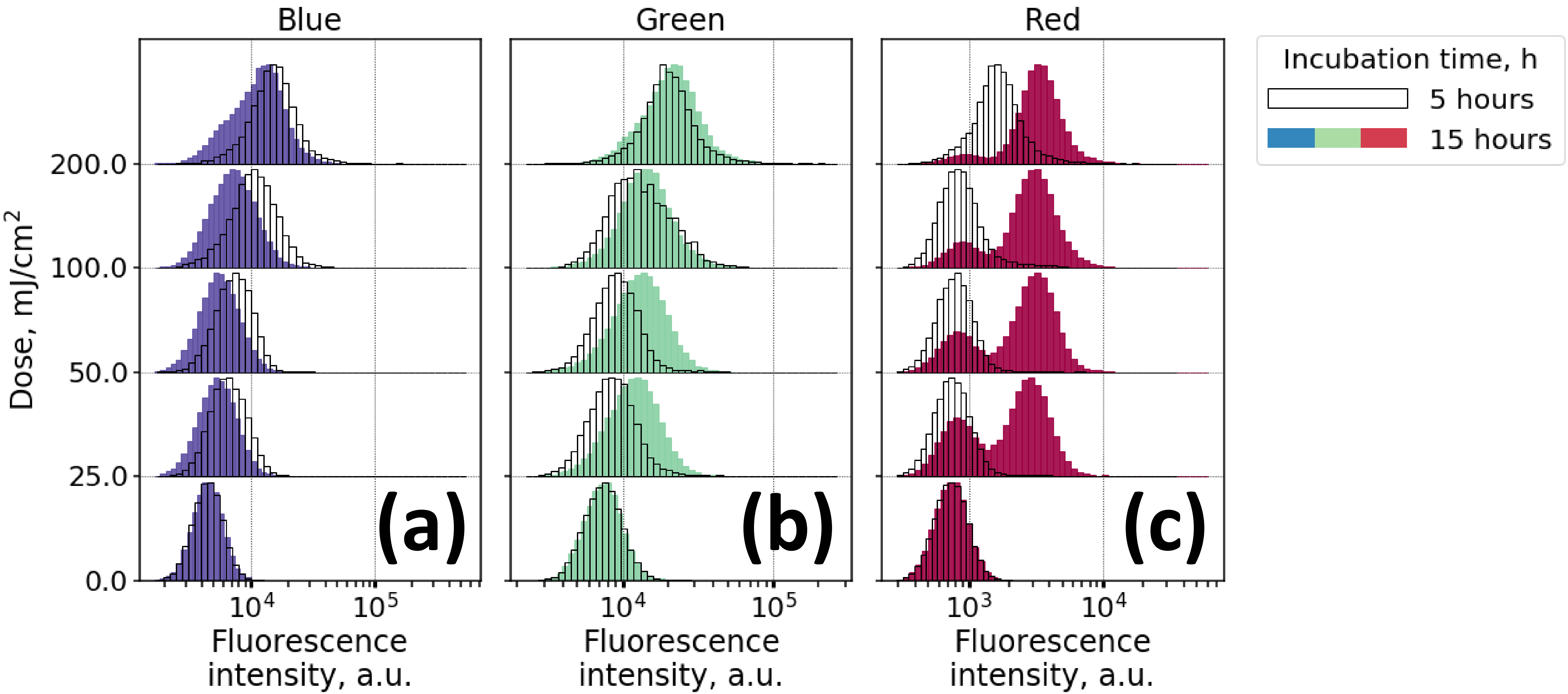

Figure S6. Analysis of flow cytometry data on HaCaT keratinocytes after UV irradiation (λ = 254 nm, intensity 10 mW/cm^2) at different dose. Fluorescence (FL) intensity values in different spectral channels: (a) – Blue FL; (b) – Green FL; (c) – Red FL. Raw data without normalizing on scattering.

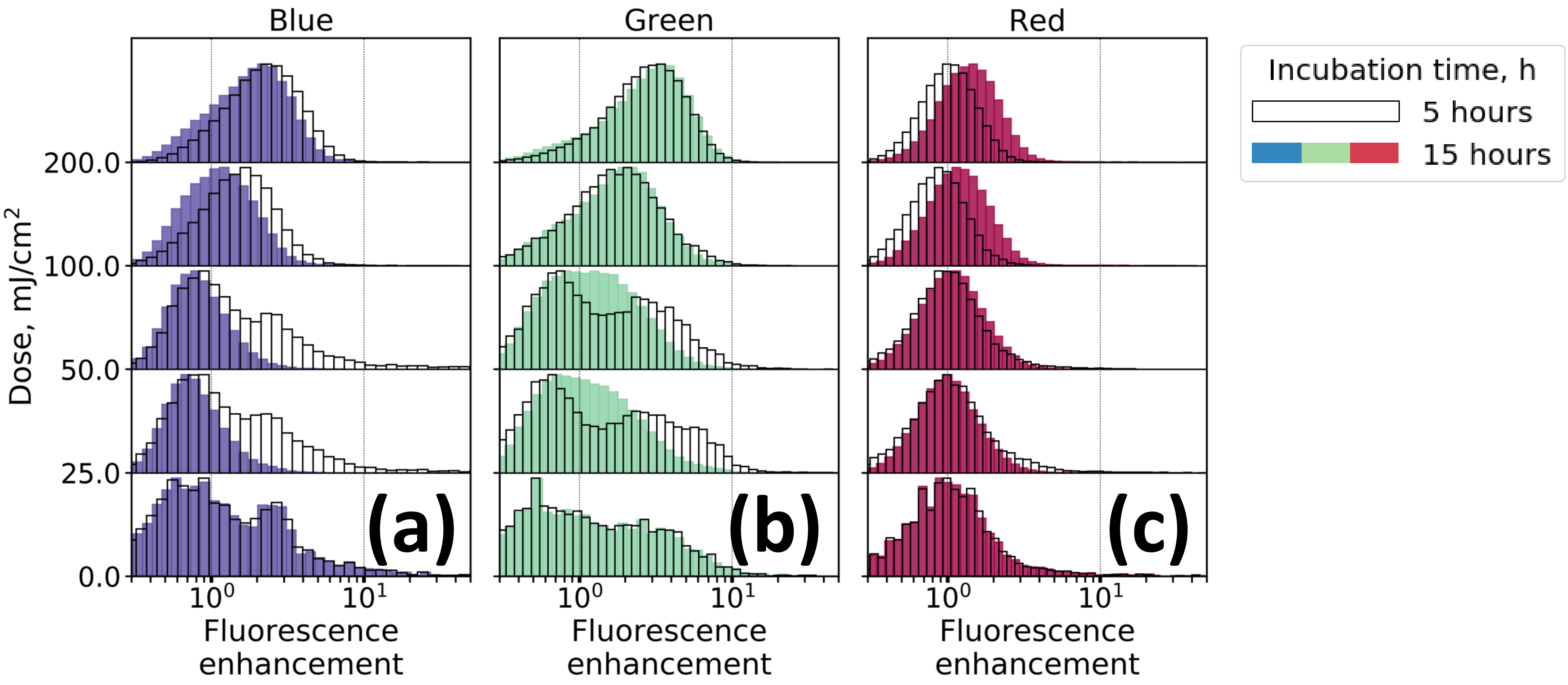

Figure S7. Analysis of flow cytometry data on HaCaT keratinocytes after UV irradiation (λ = 254 nm, intensity 10 mW/cm^2): Fluorescence enhancement in debris. Note that almost non fluorescence enhancement observed after UV for different incubation time after irradiation.

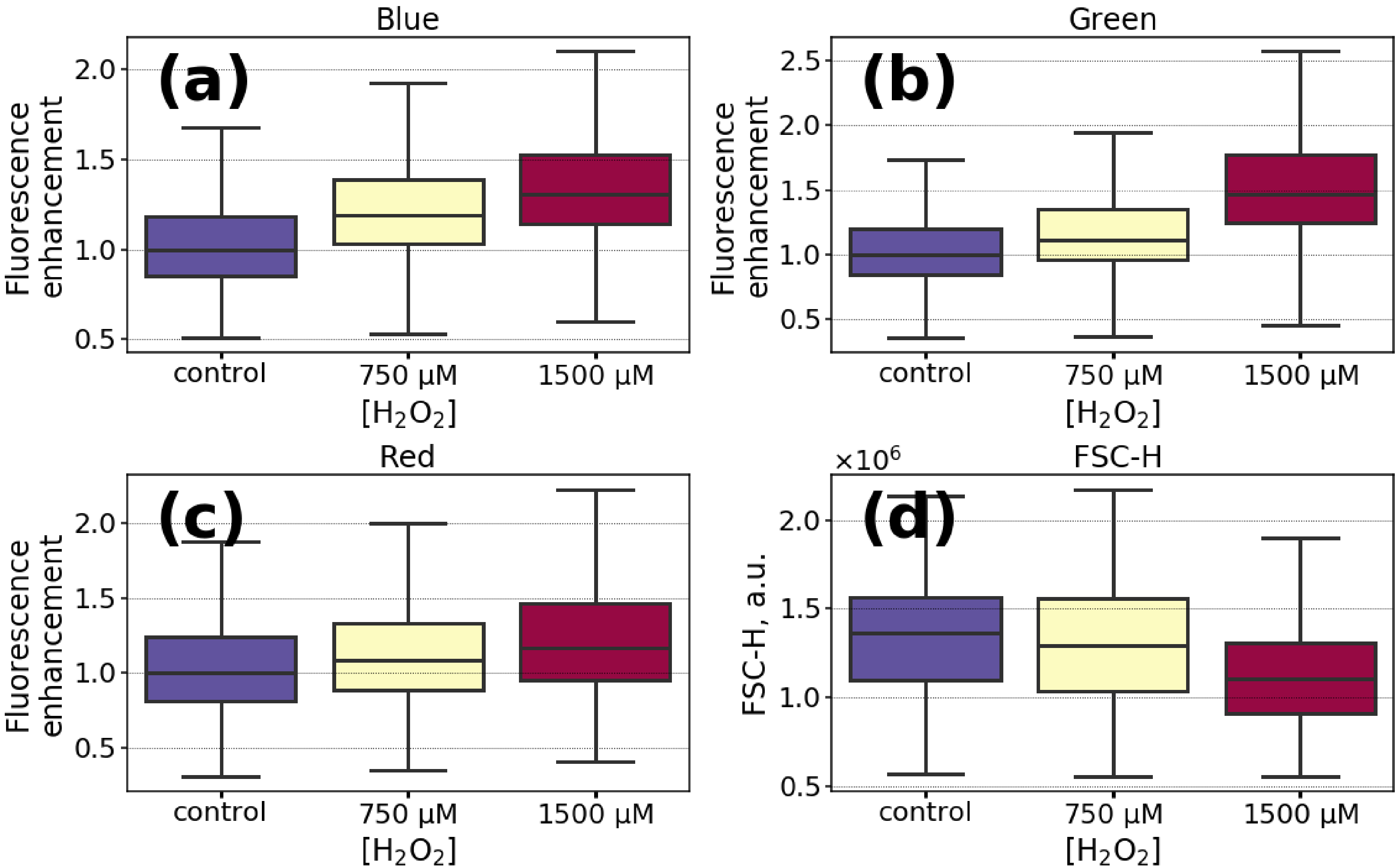

Figure S8. Flow cytometry data (Fluorescence (FL) enhancement factors and forward light scattering FSC) of HaCaT keratinocytes in different spectral channels after 19 hours of cells incubation under standard conditions at the presence of peroxide H_2O_2 at different concentration: (a) – Blue FL; (b) – Green FL; (c) – Red FL; (d) – FSC at 488 nm illumination. The effect of H_2O_2 on AF of keratinocytes was studied by addition of the peroxide stock solution into the cells growth media (DMEM) to achieve desired concentration (750 and 1500 µM). After that the cells were incubated under standard conditions during 19 hours.

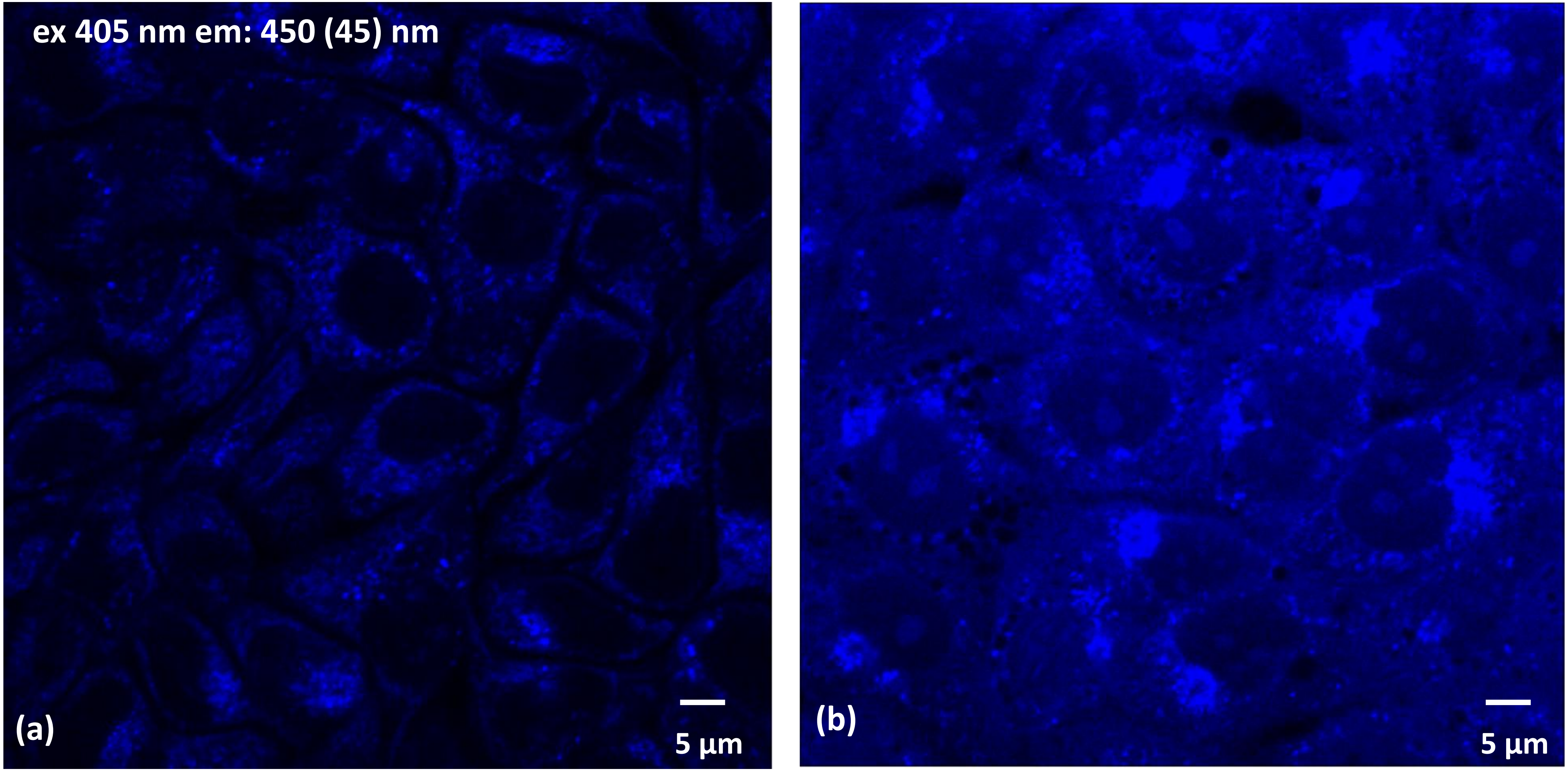

Figure S9. Autofluorescence confocal imaging of HaCaT keratinocytes monolayer (ex = 405 nm / em = 450 (50) nm): (a) - control intact sample; (b) – within 5 hours after UV irradiation (λ = 254 nm, intensity 10 mW/cm^2) at 100 mJ/cm^2.

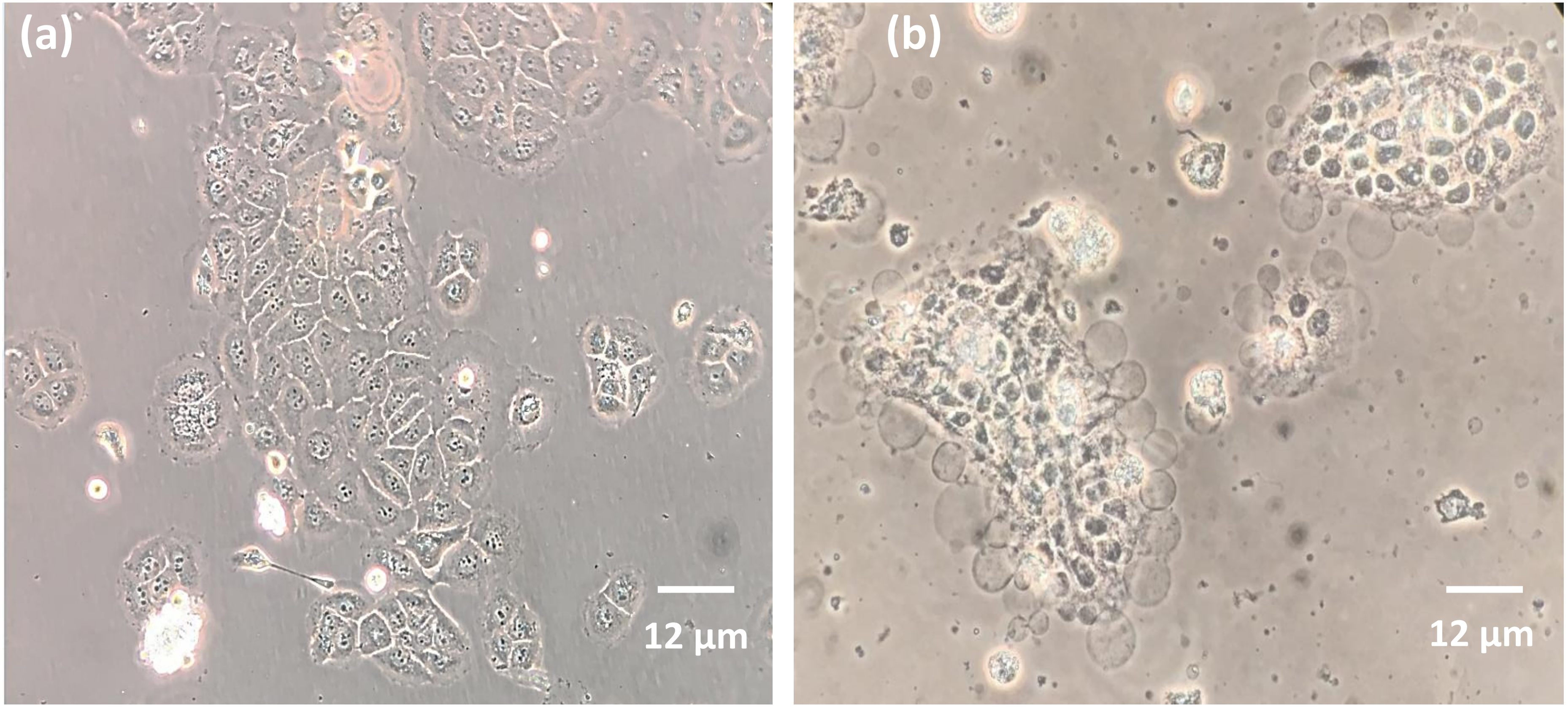

Figure S10. Optical microscopy images of HaCaT keratinocytes: (a) control intact non-irradiated sample; (b) – within 5 hours after UV-irradiation (λ = 254 nm, intensity 10 mW/cm^2) at 100 mJ/cm^2. HaCaT cell line was kindly presented by laboratory of cellular biology of N.K. Koltzov Institute of Developmental Biology of Russian Academy of Sciences, Moscow, Russia.

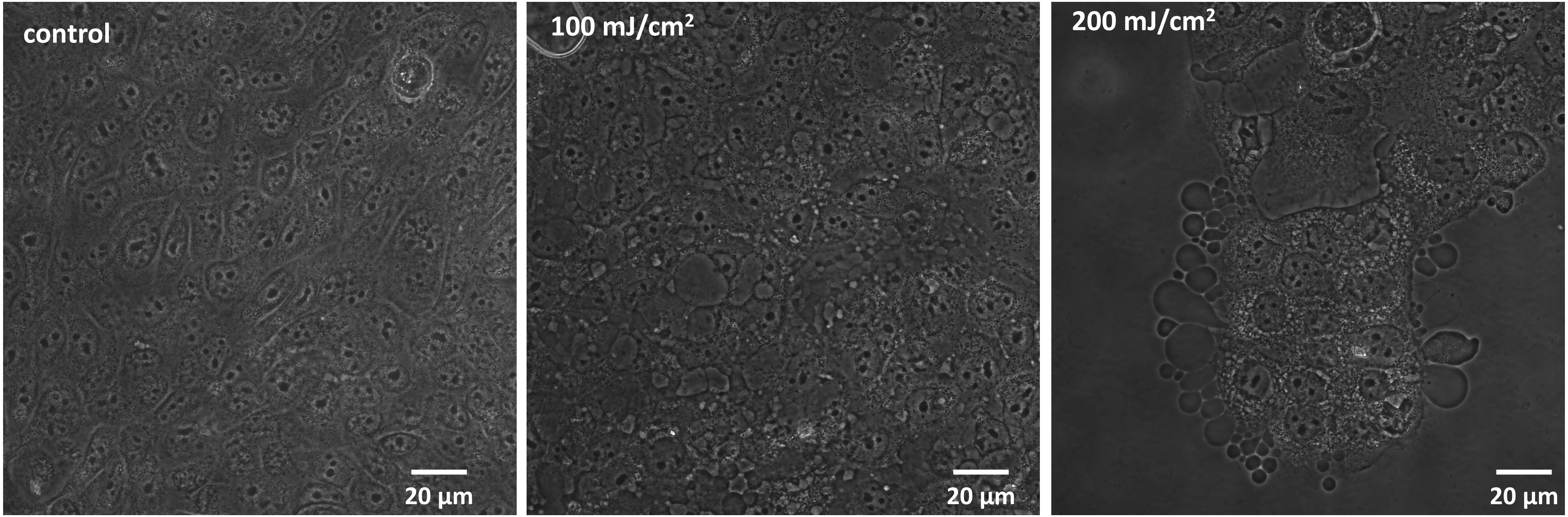

Figure S11. Optical microscopy images of HaCaT keratinocytes sample within 5 hours after UV irradiation ($\lambda = 254$ nm, intensity 10 mW/cm^2) at different doses.

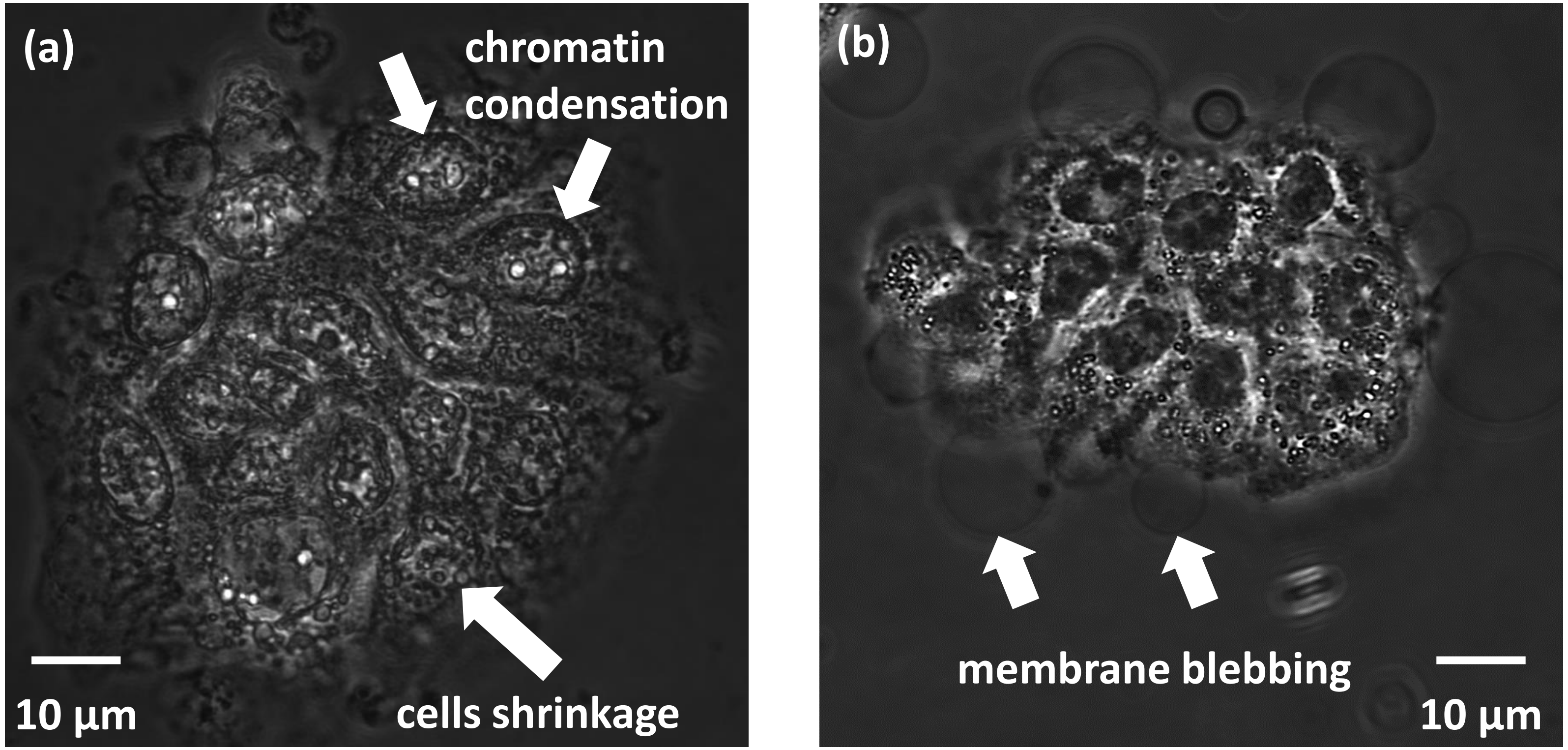

Figure S12. Observed morphological alterations of HaCaT keratinocytes under UV irradiation ($\lambda = 254$ nm, intensity 10 mW/cm^2) at high dose (100 mJ/cm^2).

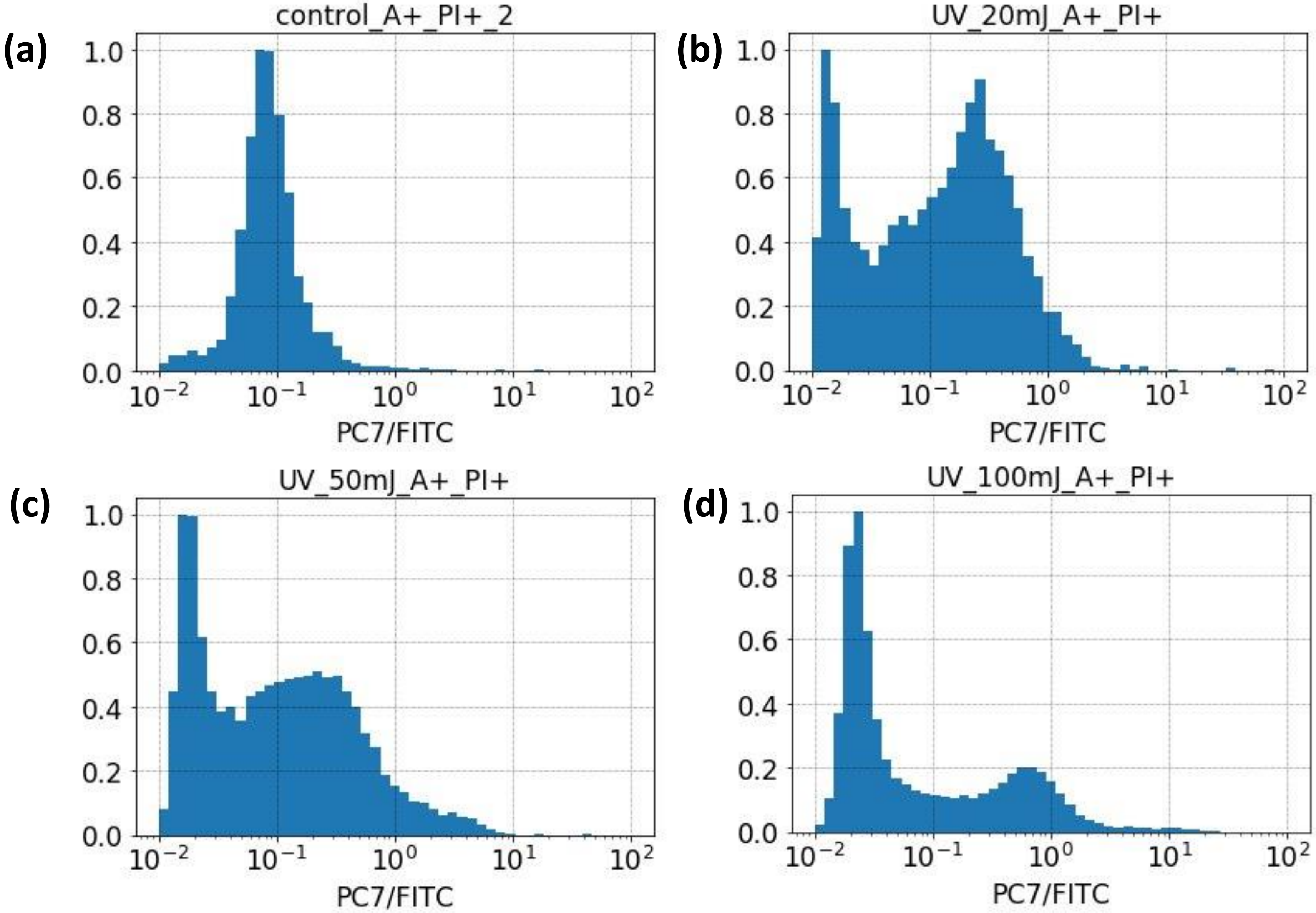

Figure S13. Flow cytometry data of apoptosis\necrosis processes in HaCaT keratinocytes samples after UV irradiation (λ = 254 nm, intensity 10 mW/cm^2) at different doses: (a) – control non-irradiated cells; (b) – 20 mJ/cm^2; (c) - 50 mJ/cm^2; (d) - 100 mJ/cm^2. The assay was performed using Annexin V/propidium iodide staining. Annexin V binding was estimated in FITC (ex=488 nm/ em=525 (40) nm) channel and propidium iodide staining was measured in PC7 (ex=488 nm/ em=780 (60) nm) channel.

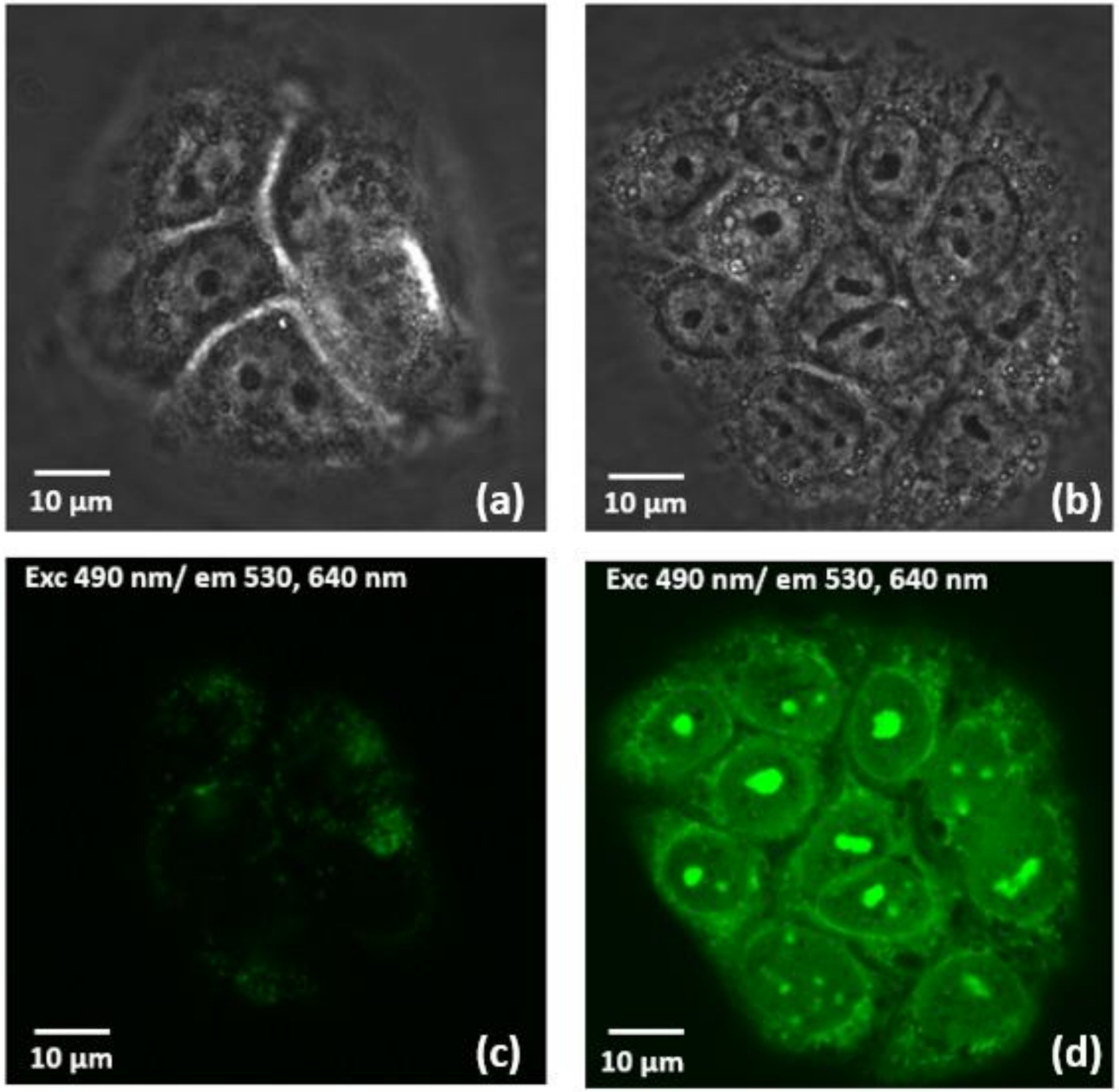

Figure S14. Confocal imaging of Singlet Oxygen Sensor Green (SOSG) probe staining of HaCaT keratinocytes: (a), (c) – control non-irradiated cells; (b),(d) – within 5 hours after UV irradiation (λ = 254 nm, intensity 10 mW/cm^2) at 100 mJ/cm^2.

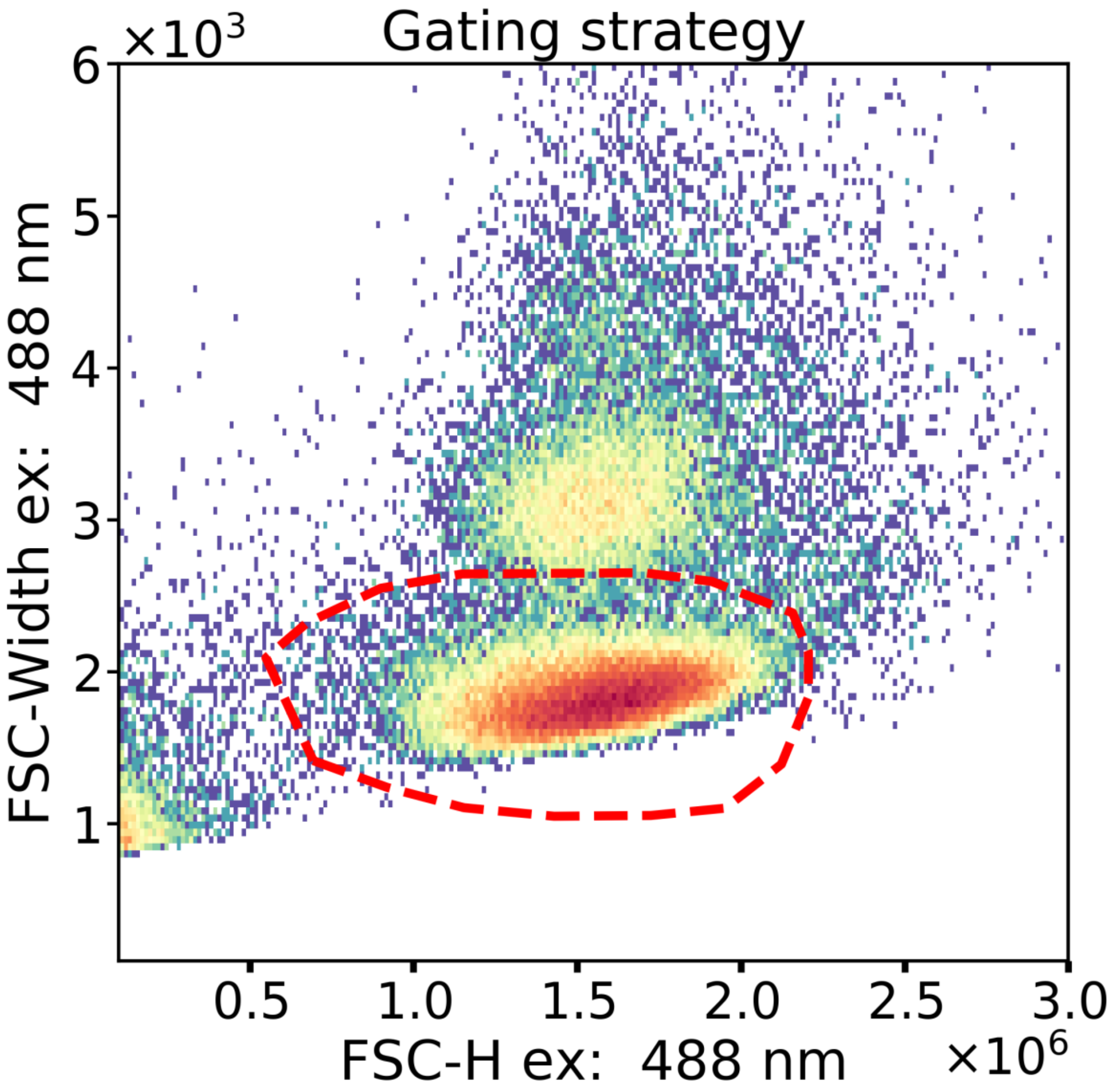

Figure S15. Gating strategy to determine the cluster of single cells during flow cytometry data analysis.

UV-irradiation dose D (mJ/cm²)	Conditions of irradiation of HaCaT keratinocytes					
	Lamp intensity I (mW/cm²)	DMEM absorption coefficient μ (cm^{-1})	Irradiation duration t (s)	Radius of Petri dish R (cm)	Volume of DMEM above cells layer V (µl)	Height of DMEM layer above cells layer h (cm)
25	10	10	30	1.75	1000	0.10
50	10	10	30	1.75	750	0.08
100	10	10	60	1.75	750	0.08
200	10	10	120	1.75	750	0.08

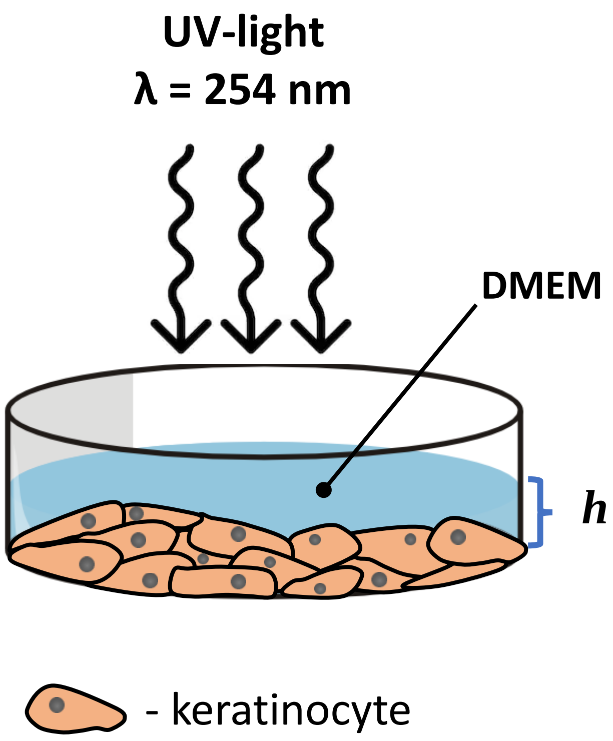

$$h = V/_{\pi R^2}$$

$$D = I \times t \times 10^{-\mu \times h}$$

Supplementary Table. Conditions of the UV-irradiation (λ=254 nm) of HaCaT keratinocytes, illustration of the design of UV-irradiation and the formula of irradiation dose (***D***) calculations.
